# Simulating an Infection Growth Model in Certain Healthy Metabolic Pathways of *Homo sapiens* for Highlighting Their Role in Type I *Diabetes mellitus* Using Fire-Spread Strategy, Feedbacks and Sensitivities

**DOI:** 10.1371/journal.pone.0069724

**Published:** 2013-09-09

**Authors:** Somnath Tagore, Rajat K. De

**Affiliations:** 1 Department of Biotechnology and Bioinformatics, Padmashree Dr. D. Y. Patil University, Navi Mumbai, India; 2 Machine Intelligence Unit, Indian Statistical Institute, Kolkata, India; Indian Institute of Science, India

## Abstract

Disease Systems Biology is an area of life sciences, which is not very well understood to date. Analyzing infections and their spread in healthy metabolite networks can be one of the focussed areas in this regard. We have proposed a theory based on the classical forest fire model for analyzing the path of infection spread in healthy metabolic pathways. The theory suggests that when fire erupts in a forest, it spreads, and the surrounding trees also catch fire. Similarly, when we consider a metabolic network, the infection caused in the metabolites of the network spreads like a fire. We have constructed a simulation model which is used to study the infection caused in the metabolic networks from the start of infection, to spread and ultimately combating it. For implementation, we have used two approaches, first, based on quantitative strategies using ordinary differential equations and second, using graph-theory based properties. Furthermore, we are using certain probabilistic scores to complete this task and for interpreting the harm caused in the network, given by a ‘critical value’ to check whether the infection can be cured or not. We have tested our simulation model on metabolic pathways involved in Type I *Diabetes mellitus* in *Homo sapiens*. For validating our results biologically, we have used sensitivity analysis, both local and global, as well as for identifying the role of feedbacks in spreading infection in metabolic pathways. Moreover, information in literature has also been used to validate the results. The metabolic network datasets have been collected from the Kyoto Encyclopedia of Genes and Genomes (KEGG).

## Introduction

An important aspect of metabolic pathway analysis is studying the impact of infections or disease spread in healthy metabolic pathways. Tackling the growth of infection in a healthy metabolic pathway as well as curing it simultaneously is rather complex. Let us consider a scenario, wherein, a metabolite in a healthy metabolic network becomes infected due to some mutation or external perturbation. Moreover, a metabolite is said to be infected if its formation, in a metabolic pathway, is somehow impaired. The major problem lies in tracking the progress of this infection to other non-infected neighboring metabolites and understand the nature of this spread [1]. The result of such an infection may further give rise to in improper production of certain metabolites leading to improper functioning of the entire metabolic pathway. In case of a healthy metabolic network, tracking this path is very difficult. The reason is that prior probability of a healthy metabolite to be infected is difficult to predict. Moreover, once the metabolite is infected, it can infect its neighbors with an infection rate, and can also be cured with a curing rate. Once cured or healthy, the metabolite is again prone to the infection. However, both infection and curing process may occur independently [2].

In this study we have implemented the forest fire strategy for analyzing the infection spread in a healthy metabolic pathway. The classical forest fire algorithm suggests that when fire erupts in a forest, it spreads to its surrounding trees, resulting in their further burning. At each step of this process, the burning trees have a probability of staying on fire and burning out, whereas the surrounding trees also have a probability of ignition [3]. This fundamental idea can be taken into consideration for studying the spread of infection in metabolic networks that are represented as directed graph format. Infection can be caused in the healthy metabolites via an infected metabolite, which can spread further and hence can be used to study the harm caused to the overall metabolic network [4]. In such cases, there is a possibility that either the metabolites are infected, safe or cured. Also, infections can be caused either by its nearest neighbors or its next-nearest neighbors. We have constructed a simulation model which can be used to study the infection caused in a network from infection initiation, to spread and ultimately combating it. Moreover, we have used certain probabilistic scores to complete this task that results in interpreting the harm caused in a healthy metabolic network, given by certain ‘critical value’ for checking whether the infection can be cured or not [5]. Thus, any harm caused to any metabolite in the pathway can provide a clear picture of the overall infection spread. But, the difference between a metabolic pathway and forest is that in the former, there are no direct contact links, wherein contacts are linked as reaction links. Also, there are many other factors involved, such as, feedback links, presence of topological units, to name a few, which must be considered for predicting the cause and nature of the path of infection spread. We have used strategies such as feedback link prediction, sensitivity approaches for handling such instances (discussed in details later).

Recent literature shows that some basic disease models have been developed to analyze the spread of certain diseases in real-world networks. These models describe the susceptibility, infection scenario and recovery rates of populations from a particular disease. In all these models information related to infection progress is not taken into account because of the differences in response among individuals in a specific population. Moreover, based on the epidemiological studies of individuals, two standard models, namely, susceptible-infectious-recovered (SIR) and susceptible-infectious-susceptible (SIS), have been proposed (discussed in section ‘Methodology’) for analyzing the study of disease spread in populations [6]. These models work on determining the source of infection and then linking each infected individual to one another, as well as to a variable number of others to whom they transmitted the disease. It generated a network of individuals consisting of all the links through which infection spreads in course of a single disease outbreak. Furthermore, some contact-tracing approaches were also developed to identify all potential transmission contacts from a source individual. These approaches identified a new set of individuals who might have the tendency to get infected from some already infected individuals. It has been applied in cases of sexually transmitted diseases (STDs) where a contact is most easily defined. But, all these network-based studies are limited by fact that there is no simple way to relate the sensitivity of the results to the details of the network structure. We have studied the path of infection spread by developing an algorithm based on SIS model (discussed in section ‘Methodology’) [7].

To understand the feature of fire spread, we have selected two strategies, first, based on quantitative studies, and second, based on graphs. The first strategy implements the fire spread using mathematical models and expressions. We have used ordinary differential equations (ODEs) for this purpose. We use ODEs for representing the complete metabolic pathway, its constituents as well as their ongoing interactions. As these metabolic processes are dynamic in nature, modeling them using ODEs is extremely useful [8]. The second strategy, based on graph theory, considers connectivity patterns and other structure-based properties among metabolites for implementing the fire spread in the metabolic pathway. Here, we represent the metabolic pathways in the form of directed graphs. Since, we are proposing a computational strategy for understanding the fire spread process, it is essential to validate it biologically. The reason is that the strategy may work *in-silico* but may fail when implemented on a real dataset. For this purpose, we have modeled and analyzed a prevalent property of metabolic pathways, namely, occurrence of feedback reactions and studied its role in disease spread [9]. Again, for this analysis, we have proposed a quantitative method. Furthermore, we have found that certain metabolites play a key role in disease spread and combat. For biological validation, we have used the technique of sensitivity analysis to understand the nature and property of these metabolites and their role in disease spread. Sensitivity analysis is a mathematical implementation of understanding the systematic change in the metabolic pathway due to perturbations, both internal and external (discussed in details in section ‘Methodology’) [10].

For testing our simulation tool, we have selected Type I *Diabetes mellitus* in *H. sapiens*. *Diabetes mellitus* is a metabolic disorder of multiple aetiology that is characterized by chronic hyperglycaemia affecting carbohydrate, fat and protein metabolism, which results from improper insulin production. *Diabetes mellitus* affects in various manners, which include long-term damage, dysfunction and failure of multiple organs. Furthermore, certain genes play a vital role in the development of *Diabetes mellitus*. To date, more than 250 candidate genes have been investigated, and results have shown a very high variability in gene association with *Diabetes mellitus* [11]. But, it is yet to be identified all the gene mutations that put a person at risk for *Diabetes mellitus*. Even if mutations are known, some investigations have found that people with low risk genes can still develop *Diabetes mellitus*. Moreover, it has been observed that the combination of susceptible genes and environmental factors may initiate this disease process that is associated with the formation of an autoimmune response to the insulin-producing cells. This autoimmune reaction is reflected by the presence of antibodies against prominent antigens in the pancreatic 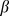-cells [12].

Type I is usually characterized by the presence of anti-Glutaric acid decarboxylase (GAD), islet cell or insulin antibodies which identify the autoimmune processes that lead to 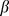-cell destruction. The insulin gene (INS) is the second well established susceptible locus in *Diabetes mellitus*. It contributes about 10% toward *Diabetes mellitus* susceptibility [13]. We have analyzed the onset of Type I *Diabetes mellitus* in *H. sapiens* by studying the role of GAD and INS genes in metabolic pathways involving Type I *Diabetes mellitus* from a Systems Biology perspective. For this purpose, we have used the SBML format of metabolic pathway datasets under Type I *Diabetes mellitus* of *H. sapiens* for our study. We have downloaded the metabolic pathway datasets which have shown role in the expression of GAD and INS genes, known from Kyoto Encyclopedia of Genes and Genomes (KEGG) [14], and used KEGG2SBML tool for converting them in SBML format [15].

## Materials and Methods

Here we describe the method we have developed for implementing fire spread in healthy metabolic networks in *H. sapiens*, analyzing their tendency to become infected giving rise to Type I *Diabetes mellitus*. We have collected the metabolic pathway datasets involving GAD and INS genes from Kyoto Encyclopedia of Genes and Genomes (KEGG). One of the criteria for handling our algorithm requires input to be given in Systems Biology Markup Language (SBML) format. We used the KEGG2SBML tool for converting the metabolic pathway datasets from KEGG to SBML format [15]. Systems Biology Markup Language (SBML) is an XML-based language for representing biological network-based models. Any biochemical reaction in a metabolic pathway can be represented into a number of XML-based elements like reactant species, product species, reactions, stoichiometric rates, and some other parameters necessary for the reactions to occur [16]. Similarly, a network of reactions can also be represented in the same manner. An SBML representation consists of certain standard modules, like *compartment* which acts as a container of finite volume where reactions take place, *species* which represents an entity that takes part in a reaction, *reaction* which describes some transformation process converting one or more species, *parameter* which describes a quantity taking part in a reaction process, *unit definition* which specifies a name of a unit used in the expression of quantities in a reaction model, and *rule* which acts as a mathematical expression that is added to the model equations constructed from the set of reactions.

The complete methodology is divided into *five* steps, which are performed one after another in successive manner. The *first* step is quantitative formulation of the metabolic pathways using ordinary differential equations (ODE), which deals with conversion of the entire metabolic pathways in the form of ODEs. This is one of the preliminary aspects of quantitative modeling. The *second* step is fire spread analysis, which involves modeling the fire spread mathematically using information from the *first* step and implementing it into the healthy metabolic pathway. The *third* step is handling the presence of feedback reactions, analyzing their role in fire spread and combat. This step has two sub-steps, namely, modeling feedback reactions and identifying them in the metabolic pathways. The *fourth* step deals with analyzing the sensitivity threshold of metabolic pathways against this fire spread. We performed both local as well as global sensitivity analysis. The *fifth* and last step is damage analysis, which calculates the extent of infections that spread throughout the metabolic pathway and the metabolites that remain uneffected/healthy or which have become cured after becoming susceptible to infection attack. For implementation purpose, we selected glutamate metabolism for further explanation.

### Quantitative formulation of metabolic pathways using ordinary differential equations (ODEs)

Here, we have used ODEs to model the entire glutamate metabolism in terms of the metabolites participating in various reactions as shown below. Our intention of performing this step was to model the spread of infection mathematically, for which initial structure of the healthy glutamate metabolism needs to be converted into ODE form. For this purpose, we assumed some notations, namely, 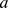 to 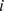, which represent genes, and 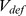, default volume of the compartment. The unit-wise representation of the metabolites is 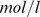. The other parameters involved are the kinetic parameters whose initial values are assumed to be 1 [17]. The ODEs of glutamate metabolism in terms of its participating non-pool metabolites are as given below.
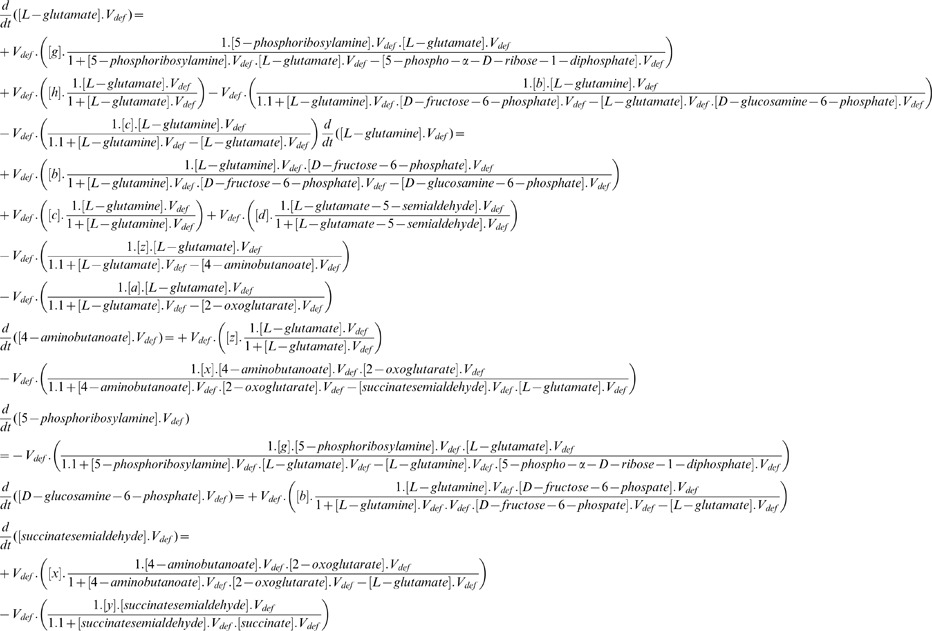


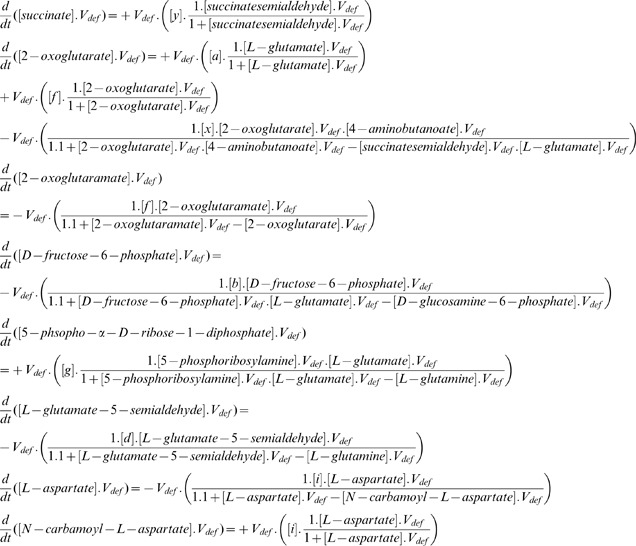



Now, considering the ODE for 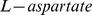, we observe that it participates in reaction 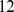 only, where it acts as a reactant species. Thus, 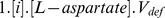 in ODE stands for reactant contribution whereas 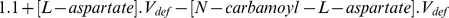 stands for product as well as contribution of 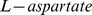 associated with 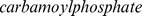 as reactants [18]. Similarly, the ODE representations of other metabolites have also been constructed in identical manner. Thus, modeling metabolic pathways quantitatively helped us to understand the initial structure of healthy metabolic pathways before being subjected to disease spread. Also, now we are in a position to understand the spread of disease in this healthy metabolic pathway and represent it mathematically for further simulation studies.

### Fire spread analysis

**Figure 1:**
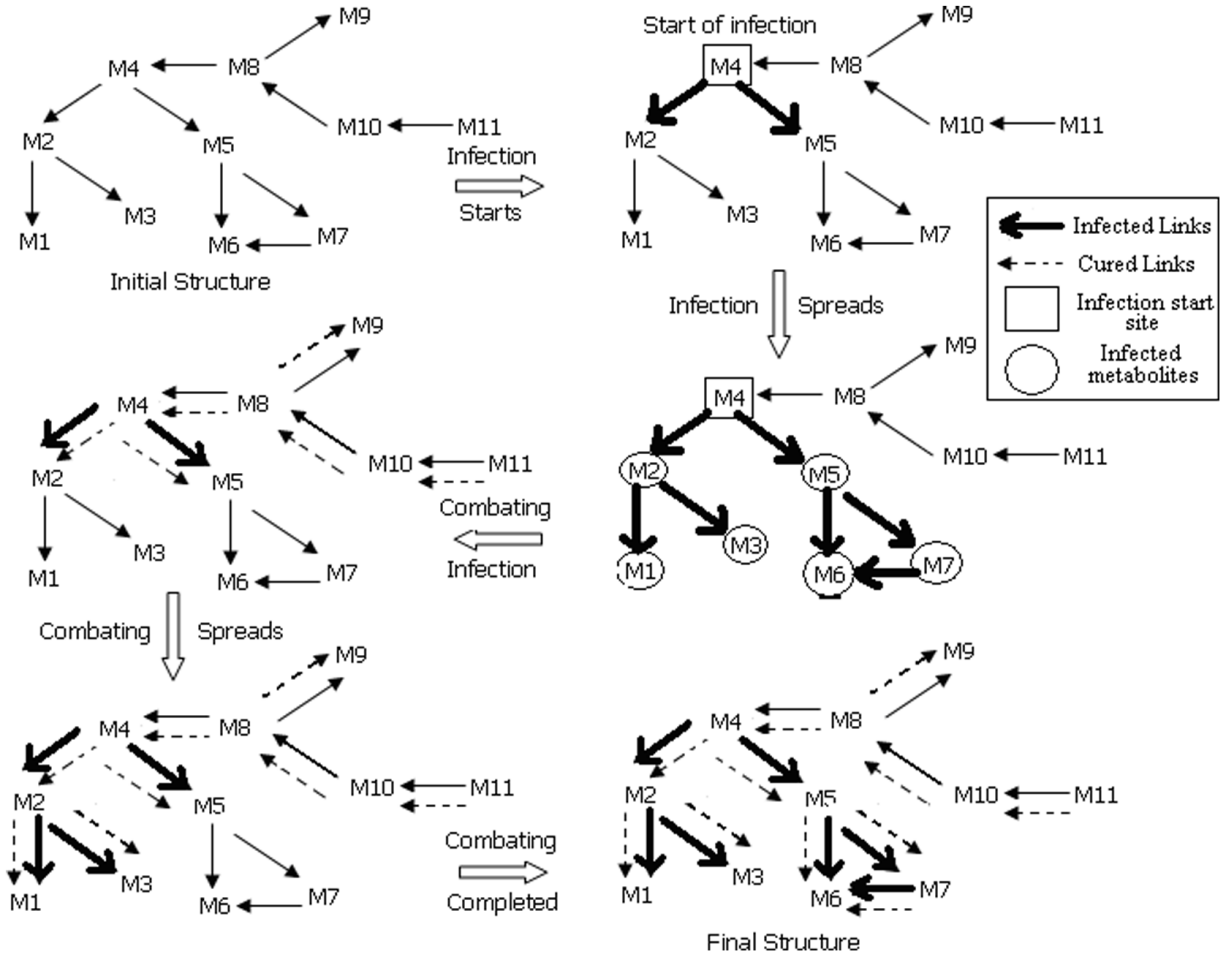
Analyzing the spread of infection and combat process in a hypothetical metabolic network; nodes represent metabolites, edges represent reaction links, bold lines signify infection, dotted lines signify combat.

One of the widely analyzed epidemic models is the susceptible-infected-removed or SIR models [16]. In this work, we implemented the SIR model with certain modifications in four healthy metabolic pathways of *H. sapiens* to analyze their susceptibility for T1D. The original SIR model was first proposed by Lowell Reed and Wade Hampton Frost in 1920. It discussed the growth of an epidemic in a population of individuals, where the population is divided into three states, namely, susceptible (S), infection (I) and removed (R). Susceptible individuals are those who have higher chance of getting infected from some already infected individuals, whereas removed state corresponds to those individuals who are either dead or removed from the populations [19]. We have discussed this section under two sub-headings, namely, mathematical modeling of fire and implementation of fire spread. In mathematical modeling of fire, we discuss the various notations regarding the SIR model in the form of ODEs, which we generate from the already generated schema of ODEs of healthy metabolic pathways. The second subSection S4eals with the actual implementation of the fire spread in glutamate metabolism of *H. sapiens* for checking its susceptibility against infection spread. Fig. 1 represents the architecture of the path of infection spread in a hypothetical pathway.

#### Mathematical modeling of fire

In the previous section, we generated the ODEs for the healthy metabolites in glutamate metabolism which are still uneffected from infection spread. Now, we focus on the structure of the same metabolic pathway after it gets infected. For the same purpose, certain notations have been considered, namely, 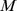 = network size, 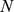 = total number of metabolites getting inserted randomly into 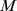, 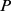 = total number of metabolites that are susceptible to infection spread, 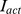 = total number of metabolites that are actively infected, 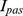 = total number of metabolites that are passively infected, 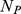 = probability that a susceptible metabolite is not cured, 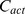 = curing rate (active), 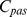 = curing rate (passive), 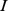 = total number of infected metabolites, 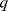 = number of infected metabolites getting degenerated, 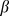 = breakdown rate, 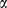 = infection rate, 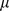 = susceptibility rate, 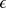 = degeneracy rate, and 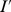 = number of cured metabolites.

First, we model the rate of change of the structure of pathway with time against possible infection attack. Thus, we have

Change in number of metabolites that are susceptible to infection attack

 = Current pathway architecture−metabolites that are infected directly or actively with a certain infection rate−susceptible metabolites getting infected with a certain susceptibility rate+metabolites that are cured actively with active curing rate+metabolites that are cured passively with passive curing rate [20]


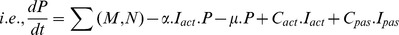
 Next, we find change in number of passive infected metabolites with time, i.e.,

Change in number of infected metabolites in passive manner

 = Number of susceptible metabolites that are actively infected−breakdown of already actively infected metabolites−curing of infected metabolites with passive curing rate−susceptibility of cured metabolites getting infected again [20] 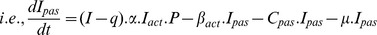 Similarly, for identifying the change in number of actively infected metabolites with time, we have,

Change in number of infected metabolites in active manner

 = Number of metabolites getting degenerated due to infection of metabolites (both susceptible and healthy) in active manner+breakdown of infected metabolites−curing of infected metabolites with a certain curing rate−susceptibility of already infected metabolites to be getting infected again−degeneration of the infected metabolites with a degeneracy rate [20] 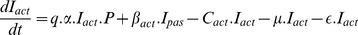 Furthermore, in case of healthy metabolic pathways, (i.e., in absence of any infection), 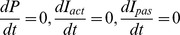 i.e., 
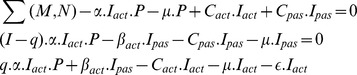
 Also, in initial state, 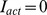, 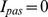, and 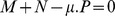. Furthermore, we have also considered the various transition events associated with the healthy metabolic pathways, after getting infected [20]. They are, 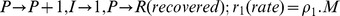 (increase in M) 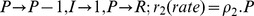 (decrease in M)
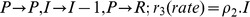
(decrease)

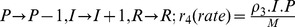
(infection)

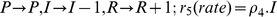
(recovery)

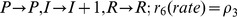
(infection)



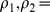
force of infection, 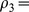
coefficient of transmission, 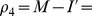
critical factor

One of the most important factors associated with this nomenclature is the critical factor (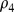), which represents the actual number of metabolites that remain uneffected after disease spread. As 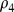 increases, the resistivity and robustness of the metabolic pathway also increases and vice-versa. The next subSection S4eals with the actual implementation of our model in glutamate metabolism of *H. sapiens*.

#### Implementation of fire spread

**Figure 2:**
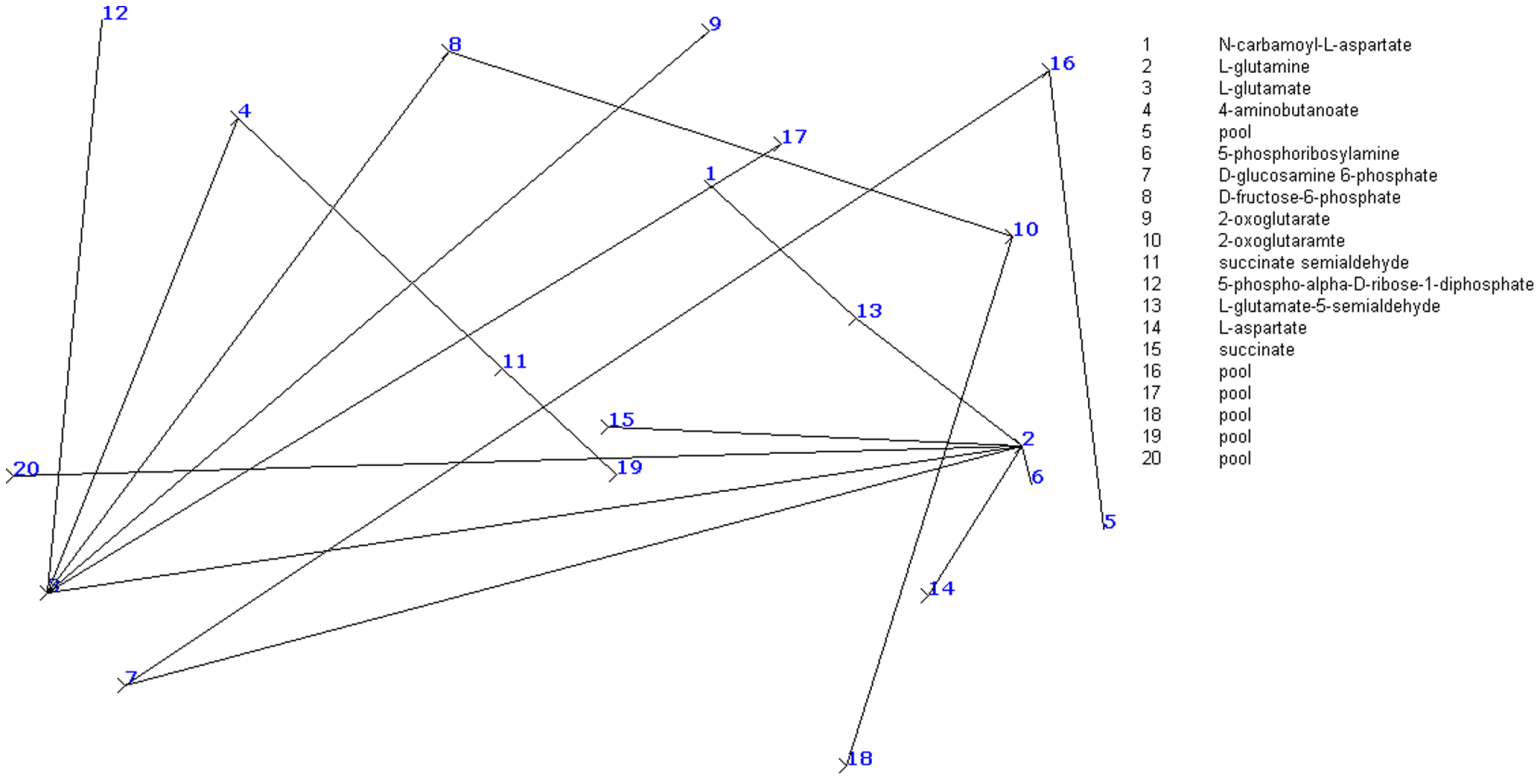
Directed graph-based representation of 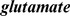 metabolism, as generated by our simulation model; nodes represent metabolites and edges represent reaction links.

For visual purpose, we represented the metabolic pathways as directed graphs, where metabolites are represented as nodes and enzymes are represented as edges [21]. Fig. 2 illustrates the glutamate metabolism in *H. sapiens* in directed graph format as generated by our algorithm.

As discussed in the previous subsection, an infected metabolite can recover back, but may again become susceptible to infection [4]. In a standard SIR model it is assumed that those which are getting immunized do not get infected again, whereas in case of our model we do not consider any metabolite to become immunized to the infection spread and consider them equally susceptible to other infected metabolites against infection [22]. In a metabolic pathway, we consider that the infection spread happens through the interconnected links among the metabolites [23]. We also consider that the most important aspect in this case is designing effective strategies for preventing and restricting the outbreak of infection. One of the effective approaches in this case is curing the infected metabolites and vaccinating the uneffected ones with a probability proportional to their connectivities, so that a greater proportion of metabolites of high connectivity are vaccinated than metabolites with low connectivity. Another strategy is specifically targeting the hub (highly connected) metabolites by vaccinating all metabolites of connectivity higher than some threshold value [6].

The graph-theory based implementation initiates with representing the input metabolic pathway (in SBML format) into a directed graph 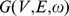, where 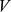 is a set of metabolites, 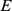 is a set of reaction links and 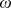 is a set of mapping functions that maps every link onto some ordered pair of metabolites (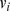, 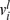). We also consider two structural attributes, namely, ‘front propagation 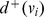
’ and ‘back propagation 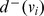
’, i.e., number of outgoing and incoming links to a metabolite. For initializing the event of fire or infection, an initial metabolite, 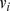 is selected that acts as the start or ignition site [7]. Thus, we consider that infection spreads from this start site to other neighboring metabolites through their connecting links. Considering glutamate metabolism, the initial pathway structure before infection spread is, 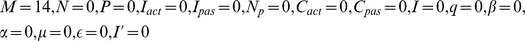. Now, fire spreads from 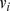, through the links connecting it to its neighboring metabolites, based upon two factors, namely, ‘Burning Probability (BP)’ and ‘Combating Probability (CP)’. The neighboring metabolites for a particular 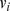 is found out from 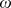. ‘BP’ is defined as the chance of a metabolite to become infected due to a neighboring infected metabolite where 
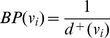
. Similarly, ‘CP’ is defined as the chance of an infected node to become cured, where 
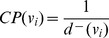
 [23].

**Figure 3:**
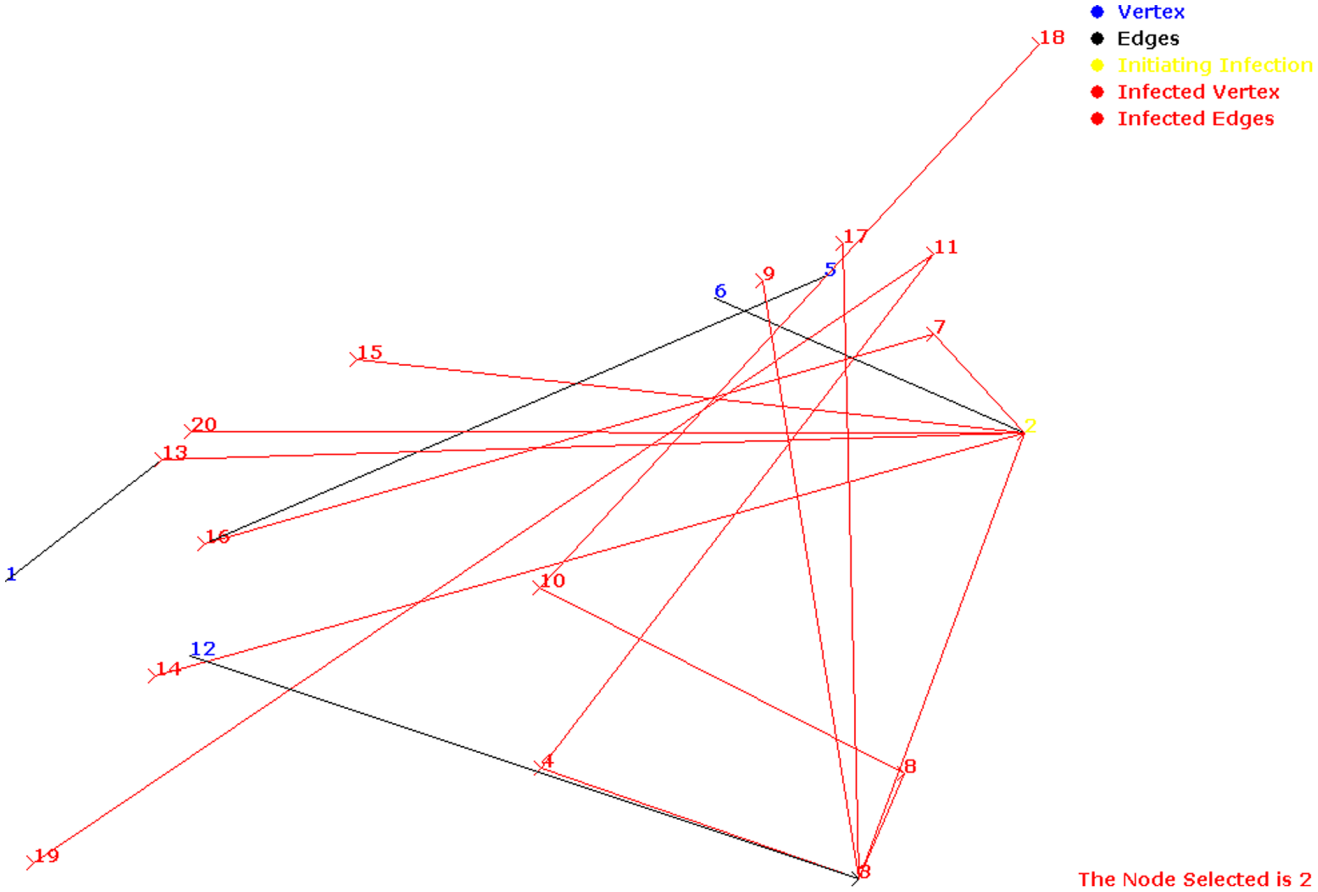
Spread of infection in glutamate metabolism in *H. sapiens* having infection start site as ‘2’.

Here, we define a metabolite as ‘infected’ when it loses its functionality, due to some mutation and external perturbation, and becomes inefficient to form a useful product. Also, a metabolite is termed to be infected if its formation, in a metabolic pathway, is somehow impaired. We assume that higher the connectivity of this metabolite, more the probability that its neighboring metabolites are infected. Also, if the metabolite is completely infected, it no longer participates in any other reaction, thus 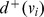 becomes 0. This state is achieved when curing fails. A ‘cured’ metabolite is that which has large number of alternate and parallel paths of its production. The reason for considering an infected metabolite to be cured is that even if it becomes infected by a path leading to its destruction, it can be produced by an alternate path. It can be found by keeping a track on the incoming links, i.e., 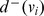. For example, in Fig. 3, BP(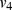) = 0.5, BP(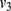) = 1.0, BP(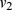) = 0.5, whereas CP(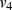) = 1.0, CP(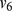) = 0.5, to name a few.

**Figure 4:**
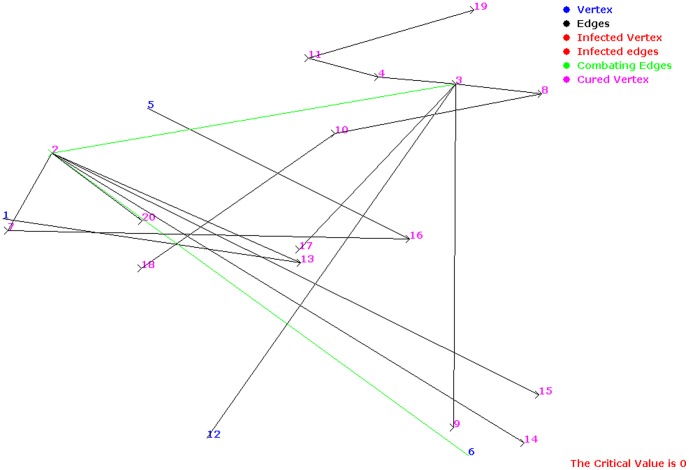
Combat process in glutamate metabolism in *H. sapiens* having infection start site as ‘3’.

Infection spreads from initial metabolite till the path ends where the value of 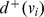 is 0 and ‘BP’ is minimum, i.e., when no further link is present connecting the infected metabolite to other healthy metabolites. All the infected metabolites are included in the set 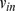. Once the infection has totally spread in the pathway, the factor taken into consideration is ‘CP’. We also store the metabolites connected to an infected metabolite in the set 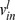. After a metabolite 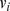 is infected, combating the infection will take place when the level of ‘CP’ is high [24]. Here, at each step the probability of combat for each metabolite changes according to the spread of infection and their bypasses. For example, BP(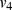) = 0.5, CP(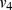) = 1.0. Thus, the probability that 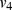 will be cured is always high. The same was the case with 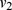, where BP(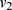) = 0.5 and CP(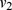) = 1.0. Thus, chances of 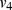 and 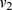 to be cured were quite high. The above simulation was with respect to the structural parameters associated with the pathway. But, biologically, only structural parameters could not be taken into consideration for simulation purpose. Thus, for adhering the biological aspect of our model, we calculated the ODE values too. Thus taking 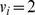, various factors associated with it were 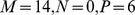 (metabolites that were not directly linked to 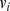 but are susceptible), 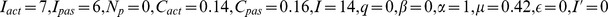. Thus, 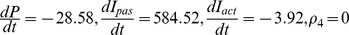. Thus, critical factor was 0, i.e., size of the pathway did not change and all infected metabolites were cured (Figs. 3, 4).

### Analyzing feedbacks

**Figure 5:**
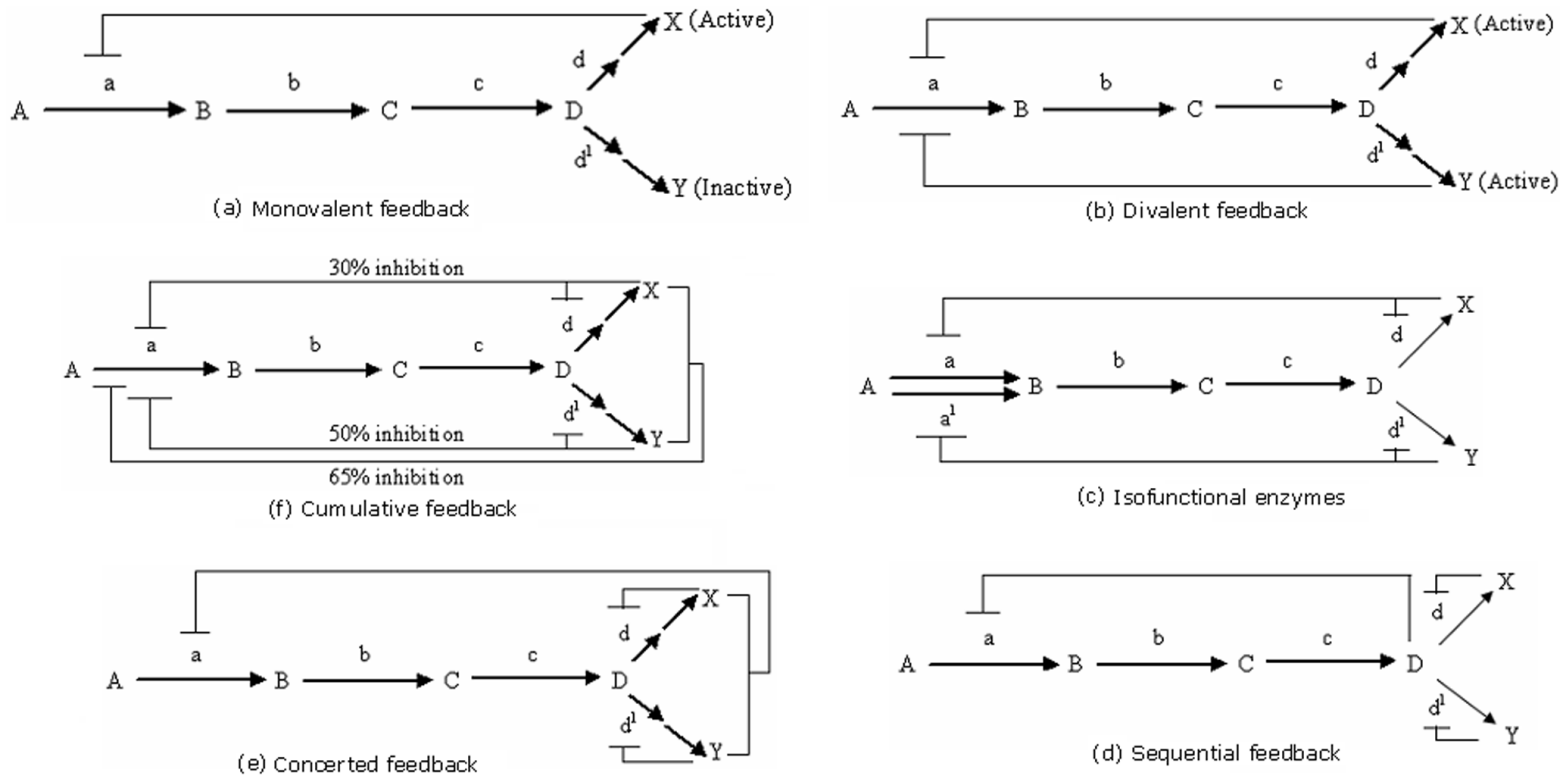
Schematic representation of certain feedback reactions that are predominant in metabolic pathways in *H. sapiens*; A–D, X and Y denote metabolites, and a–d and d' signify reaction links/genes.

**Figure 6:**
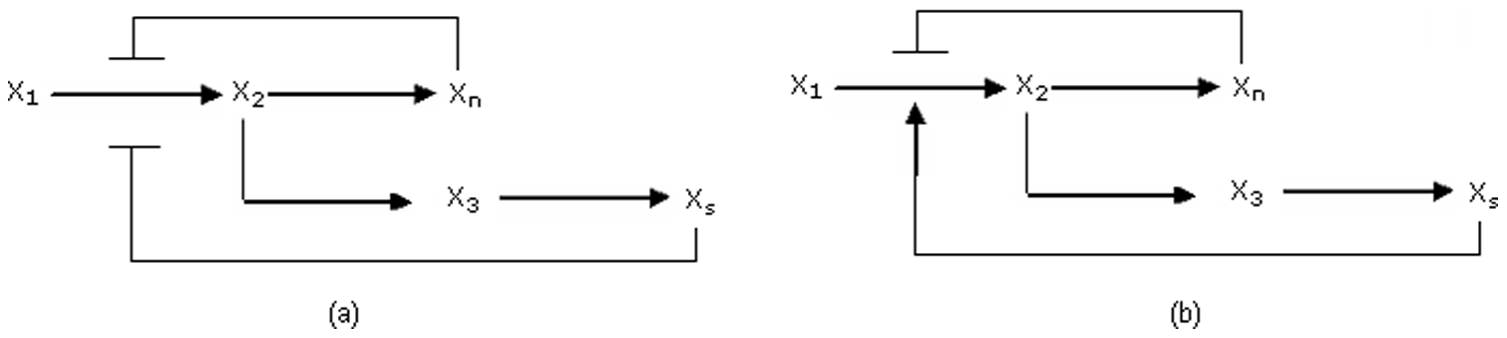
Schematic representation of a feedback loop, as found in metabolic pathways in *H. sapiens*, 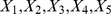 and 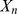 denote metabolites.

As we deal with metabolic pathways, there are various ongoing processes that we need to consider so that our simulation is successful and biologically relevant. One of the most important properties in metabolic pathways is the presence of feedback reactions, which can drastically effect their overall functionality. So, we studied the existence of feedbacks and related them to our model. This subsection explains the basic implementation strategy followed by us for identification of feedbacks [9]. The different categories of feedback reactions occurring in metabolic pathways are shown in Fig. 5, 6.

#### Modeling feedback reactions

We considered a model 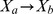 for a metabolic reaction having one substrate and one product formation. Also, the reaction is inhibited by other metabolites present in the pathway. Furthermore, we denote the reaction rate for such a reaction by 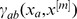, where 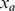 is concentration of metabolites 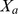 in cell and 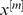 is the vector containing concentration of other metabolites inhibiting the reaction 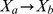. It may be noted that 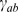 can also represent a sum of several parallel reactions that may be catalyzed by several isofunctional enzymes [25]. Moreover, larger the concentration of inhibitor, the reaction becomes much slower. Thus, we have, 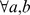 such that, 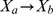, the function 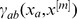 is locally Lipschitz on 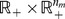, satisfying 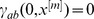, increasing in 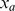 for 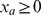 and decreasing in 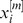 for 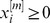. Quantitatively, we also study the presence of feedbacks using ODEs and graphs, especially, the arborescent property of graphs [25].

A directed graph is known an arborescence if, from a given node 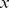 (root node), there is exactly one elementary path to some other node 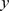. Thus, in a metabolic pathway the species involved are 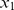, 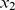, 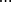, 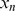 and the inhibiting reactions descend from the root 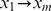, inhibited by metabolites from the sub-arborescence rooted in 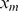, we define the mass-balance dynamical model in the form, 
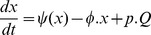
, where 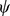 includes all reaction rates, 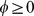 represents growth rate of the cell, 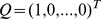, 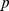 = scalar quantity, denotes constant supply rate of 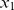 at root, 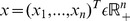, 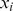 is the molar fraction of metabolite 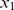 inside cell [25]. Thus, using arborescence theory, we represent the root metabolite as 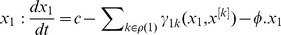 Here, 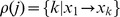 belong to the pathway. It defines the set of all metabolites that were produced by reactions having 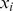 as substrate, 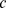 as a constant factor. Similarly, there were intermediate metabolites, 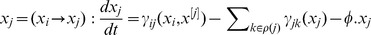 Lastly, for boundary metabolites, 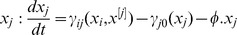


For understanding the stability of the network, 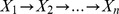, we assumed that there was only one sequential feedback inhibition [25]. Thus, the velocity of each enzymatic reaction 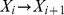 is represented by the Michaelis-Menten kinetic function, 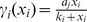 Here, 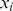 is intracellular molar fraction of 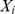, 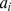 is maximal velocity and 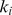 is half-saturation constant. Also the velocity of 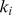 is inhibited by the last metabolite with an inhibition function, 
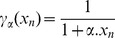
 Thus, we have, 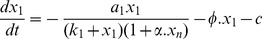 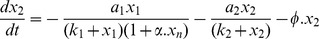 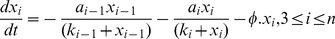 Here, 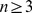, 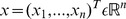, 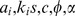 are positive constants. Now, if both 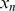 and 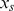 inhibits (Fig. 6) 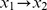, 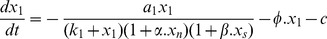 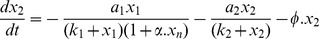 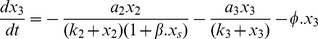 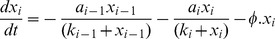


Finally, if 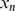 inhibits 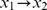 (65%) and 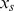 activates 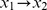 (35%), 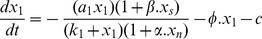 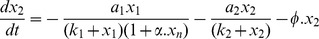 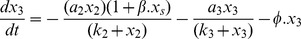


With all these notations regarding feedbacks that we generated, the ultimate problem was to define certain algorithms to identify and characterize them, so that they could be further analyzed. The next subsection highlights some algorithms that we devised to identify feedbacks in metabolic pathways.

#### Identifying feedback reactions

All the algorithms for identifying and analyzing feedback reactions were based on identifying a pattern based on graph-based properties in metabolic pathways. The property of graphs that we used were isomorphism and arborescence. The algorithm proposed by us were for identifying feedback patterns, feedback activation, feedback inhibition, monovalent link prediction, divalent link prediction, iso-functional enzyme link prediction, sequential link prediction, concerted links prediction, and cumulative links respectively.

### Symbols used

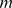
 = pattern

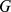
 = pathway graph

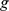
 = subgraph in 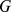

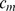
 = number of times 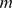 occurs in 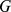

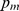
 = probability that number of time occurrence of 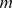 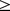 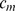

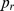
 = already defined probability threshold for the occurrence of 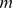

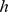
 = isomorphic subgraph of 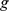

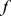
 = function defining that 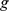 has one to one correspondence with 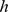

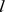
 = search graph

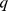
 = searching pattern corresponding to 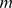

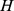
 = property value for pattern searching

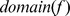
 = corresponds to 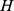

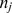
 = individual nodes in graph

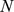
 = set of nodes in graph

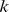
 = threshold value signifying links

### Algorithm

**Feedback pattern identification**

**for** each possible pattern 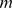 **do**

**let** 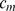 is number of times 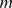 occurs in network graph 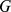


**estimate** 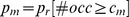


**for** each node in 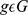 **do**

**for** each node in 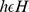 **do**

**if** 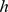 can't support 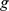 **then** **continue**

**let** 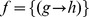



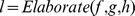


**for** 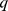 in 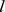 **do**

**output**

**Elaborate**(f, g, h)

**if** 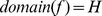 **then**

**return**[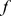]

**let** 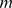 is some node in 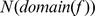


**for** each node 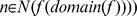 **do**

**if** adding (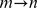) to 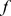 keeps 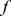 as a valid pattern, then **Elaborate**

**Activation**


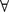
 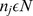


**if** 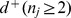 **then**

**if** link **exists** between (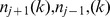) **then**

**output**

**Calculate** 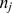 **if** 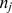 increases with time

**Inhibition**


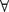
 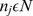


**if** 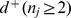 **then**

**if** link **exists** between (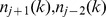) **then**

**output**

**Calculate** 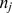 **if** 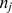 decreases with time

**Monovalent link**


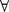
 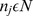


**if** 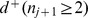 **then**

**if** 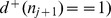 **then**

**if** link **exists** between (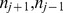) **then**

**Output: Monovalent link**

**Divalent link**


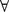
 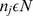


**if** 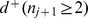 **then**

**if** 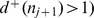 **then**

**if** link **exists** between (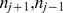) **then**

**Output: Divalent link**

**Iso-functional enzyme links**


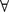
 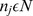


**if** 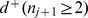 **and** link **exists** between (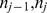) is 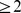 **then**

**if** 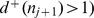 **then**

**if** link **exists** between (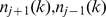) **then**

**Output: Iso-functional enzyme link**

**Sequential links**


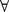
 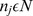


**if** 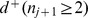 **then**

**if** link **exists** between (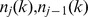) **then**

**Output: Sequential link**

**Concerted links**


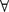
 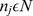


**if** 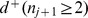 **then**

**if** link **exists** between (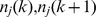) **then**

**Output: Concerted link**

**Cumulative links**


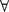
 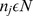


**if** 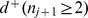 **then**

**if** link **exists** between (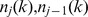) **or** between (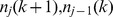) **or** between (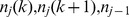) **then**

**Output: Cumulative link**

The next Section deals with analyzing another important feature of metabolic pathway that is necessary for interpreting spread of infections, known as sensitivity, which is in continuation with our studies of feedback reactions.

### Sensitivity analysis

Nesterov (1999) describes sensitivity analysis as ‘the systematic investigation of the model responses to either perturbations of the model quantitative factors or variations in the model qualitative factors’ [26]. Understanding sensitivities of metabolic pathways makes us chose those nodal points which are absolutely essential for growing the overall functioning of metabolic pathways. Sensitivities also help us to quantify the rate of change of the internal dynamics of the systems in metabolic pathways in response to external and internal perturbation, especially in case of external infection attack and spread. This section is described under two headings, namely, local sensitivity analysis and global sensitivity analysis.

#### Local sensitivity analysis

Local sensitivity analysis deals with considering changes to a single parameter at one time, by keeping others fixed. Consider a general ODE model of the form 
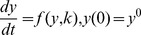
. Here, 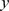 is the vector of variables, 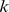 is the m-vector of system parameters and 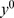 is the initial value. Thus, the effect of a small parameter change on the solution is expressed as a Taylor series expansion, 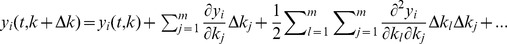 The partial derivatives 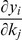 are first-order local sensitivity coefficients and form the sensitivity matrix 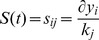. In this case, 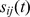 describes the effect on the 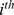 output variable at time 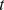 of a small change in the 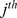 parameter around its nominal value.

Here, we used the finite-difference method for calculating the local sensitivities of the network. Using this method the model is solved at some chosen parameter point and then at some perturbed value of each parameter, 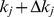 while all other parameters are held at their nominal values. The sensitivities are then calculated using the finite-difference approximation method, 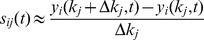. Moreover, it assumes local linearity around a nominal parameter point [27].

#### Global sensitivity analysis

Global sensitivity analysis deals with considering changes to multiple parameters at one time. Here, we used Sobol's method for performing global sensitivity analysis. In this case, given a function 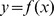 where 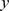 is output and 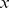 is a vector of 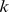 model input parameters, it can be represented in the form, 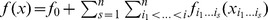, where 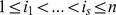. This equation is called Analysis of Variance (ANOVA) representation of 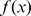 if 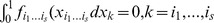


Also, we can have, 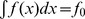 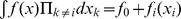 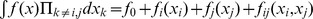 Thus, assuming that 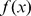 is square integrable, then all the 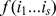 are also square integrable. So, we have 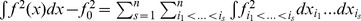


Thus, it is based on a decomposition of the variance term of increasing dimensionality. Furthermore, these partial variances are estimated using Monte-Carlo integrals and sensitivities are based on their ratio to total variances [28].

### Damage analysis

The processes of infection and curing run for a specific number of iterations, depending on the number of metabolites in the metabolic pathway. We have assigned a maximum iteration value of 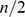, where 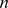 is the total number of metabolites in the metabolic pathway [5]. The reason for this threshold is that after the iteration value is 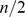, the results converge and there is no further need to continue performing further iterations. After the infection has been combated and number of iterations is completed, the critical value ‘
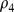
’, signifying the number of metabolites that cannot be cured, is calculated. Here, 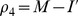, where 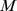 is network size and 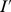 is the number cured metabolites. Three conditions can arise on the basis of calculating values of 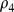. These are, 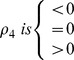


Here, 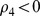 indicates that there is a certain level of noise or external metabolites which are not cured in the metabolic pathway, 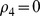 explains that all infected metabolites are cured and size of the metabolic pathway remains unchanged, whereas 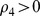 specifies that some infected metabolites are not cured completely. Thus, it highlights whether the network is completely cured or not and also helps in analyzing those metabolites, which if infected cannot be restored back, predicting the damage done to the entire metabolic pathway [29]. Moreover, for full infection removal and curing, 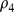 should always be 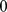.

## Results

We demonstrated the effectiveness of our method on the metabolic pathways of *H. sapiens* for Type I *Diabetes mellitus* (T1D) involving GAS and INS genes. These metabolic pathways were that of glutamate metabolism, 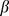-alanine metabolism, taurine and hypotaurine metabolism and butanoate metabolism. Our primary concern while working on this method was developing a framework which could be used to track the spread, prevalence and containment of any infection in normal healthy metabolic pathways of *H. sapiens*. The first step involved in this process is collecting datasets. For this reason, we searched for the metabolic pathway map of T1D for *H. sapiens* in KEGG. The genes which were explicitely involved in these pathways of *H. sapiens* (i.e., glutamate metabolism, 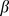-alanine metabolism, taurine and hypotaurine metabolism, and butanoate metabolism) were GAD1, GAD2 and INS respectively. The remaining genes were involved in many other networks which were not directly involved in the causal mechanism of T1D. The next step of our data collection was identifying enzymes involved in these pathways relating to T1D. This was followed by searching the biochemical reactions catalyzed by these enzymes, involved in the metabolic pathways relating to T1D. This section is described under six headings, namely, modeling metabolic pathways quantitatively, modeling infection spread in metabolic pathways, detecting feedback reactions in metabolic pathways, performing local sensitivity analysis, performing global sensitivity analysis and analyzing the damage caused in metabolic pathways due to infection spread.

### Modeling metabolic pathways quantitatively

As discussed in section ‘Methodology’, we used ODEs to quantitatively model the four metabolic pathways involved in the functioning of GAD and INS genes in T1D. This ODE model of glutamate metabolism was already discussed in section ‘Methodology’. Here, we discuss the ODE formulation of the other three metabolic pathways. The ODE representation of these pathways is present in Section S1 in File S1. For simplification purpose, the initial values of all the kinetic parameters as well as other entities, like metabolites were assumed to be 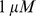 [8]. For initializing the functioning of all the reactions, the default volume, 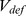 was taken into consideration. Section S1(SI) in File S1 represent the reactions present in 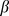-alanine metabolism, consisting of 17 reactions in total. Here, 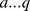 represent the genes involved in various reactions. The various kinetic parameters for reactions 1 to 17 are 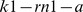, 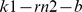, 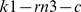, 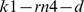, 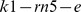, 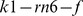, 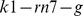, 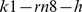, 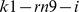, 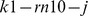, 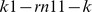, 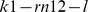, 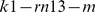, 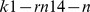, 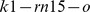, 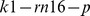 and 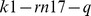 respectively. Furthermore, the kinetic parameters involved in the ODEs were different for various metabolites. For instance, in 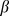-alanine metabolism, 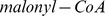 had kinetic parameters 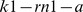 and 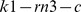, 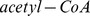 had kinetic parameters 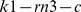 and 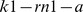, 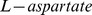 had 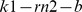, 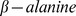 had 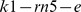, 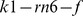, 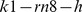, 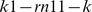, 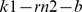, 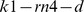, 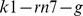, 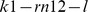, 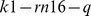, 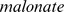 had 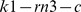, 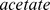 had 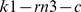, 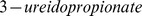 had 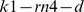, 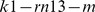, 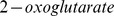 had 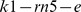, 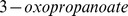 had 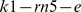, 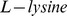 had 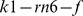, 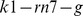, 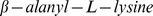 had 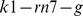, 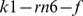, 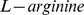 had 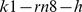, 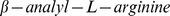 had 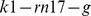, 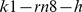, 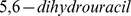 had 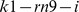, 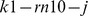, 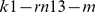, 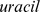 had 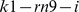, 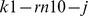, 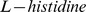 had 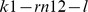, 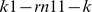, 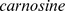 had 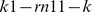, 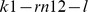, 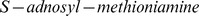 had 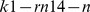, 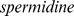 had 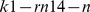, 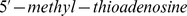 had 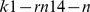, 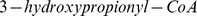 had 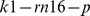, 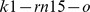, 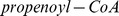 had 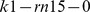 and 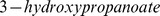 had 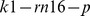 respectively.

Similarly, taurine and hypotaurine had 6 reactions (Section S1(SII) in File S1) with 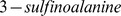 having kinetic parameters 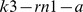, 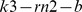, 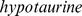 had 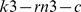, 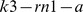, 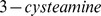 had 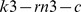, 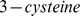 had 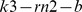, 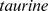 had 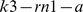, 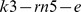, 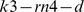, 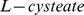 had 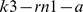, 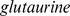 had 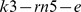, 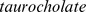 had 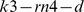, 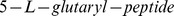 had 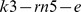, 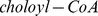 had 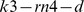 and 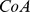 had 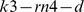 respectively. The reason for explicitly defining these kinetic parameters was that these change in accordance with the concentration of metabolites with respect to time, which plays a pivotal role for the overall functioning of the metabolic pathways.

Lastly, butanoate metabolism had 15 reactions (Section S1(SIII) in File S1)with the metabolite 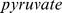 having kinetic parameters 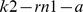, 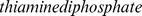 had 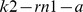, 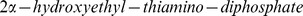 had 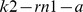, 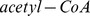 had 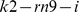, 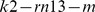, 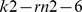, 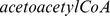 had 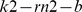, 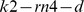, 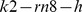, 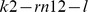, 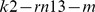, 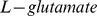 had 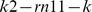, 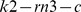, 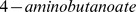 had 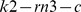, 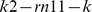, 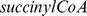 had 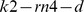, 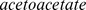 had 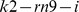, 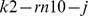, 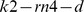, 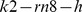, 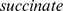 had 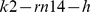, 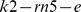, 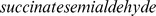 had 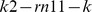, 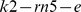, 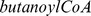 had 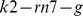, 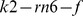, 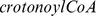 had 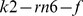, 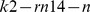, 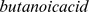 had 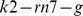, 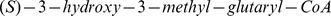 had 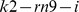, 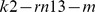, 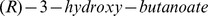 had 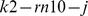, 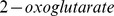 had 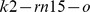, 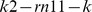, 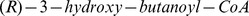 had 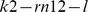, 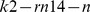 and 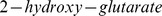 had 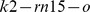 respectively [8], [30].

For validation purpose, two strategies were considered, *first*, simulating the model with step changes in input dataset as well as in time series, and *second*, comparing the predicted output with published results [31]. For the first strategy, we started with initial concentration of all the metabolites and performed two perturbations, *first*, where we progressively reduced the concentration, and *second*, where we progressively increased the concentration of the metabolites (Section G in File S1) [31], [32]. For instance, for analysing the rate of change of 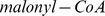 in 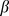-alanine metabolism, with initial concentration of 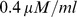, 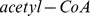 (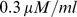), 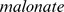 (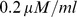) and 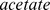 (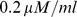), 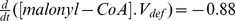 (keeping 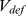 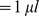, 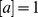, 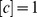). Similarly, for 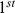 metabolite, 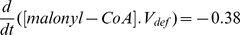, 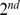 metabolite, 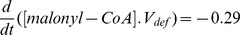, 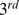 metabolite, 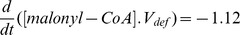 and 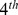 metabolite, 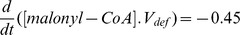 respectively. We observed that with decrease in concentration of 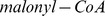, 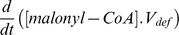 increased, whereas it decreased further when concentration was increased by 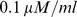 and again increased when concentration was increased by 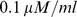. Thus, we could observe that decreasing concentration increased the rate of change and vice versa, which is predictable from the ODEs [33], [34].

Next, using the 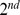 validation strategy, we found that for 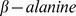 metabolism, it has been seen that when 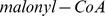 function is altered in cells, 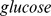-induced insulin release is impaired. Furthermore, it is already known that citrate, exported from mitochondria to the cytosol, is cleaved by ATP citrate lyase, forming 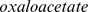 and 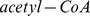, which form 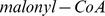 promoting fatty acid synthesis, accumulating long-chain 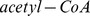 enhancing 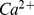 evoked inslulin exocytosis. Thus, it proved the importance of 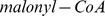 as determined by our algorithm [35]–[37].

### Modeling infection spread in metabolic pathways

The fundamental aspect of analyzing the infection spread is selecting the infection starting point. There are two methods for such selection, namely, random and targeted [5]. Random method is selecting any metabolite as infection start site without any bias. But, this does not help in disease study. The reason being, based on literature and experimental evidences, some known metabolites participating in T1D is already known. Thus, we concentrate on targeted selection, which specifically selects metabolites based on our choice (Table 1). We start our discussion with 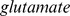 metabolism, having 15 metabolites, namely,  and 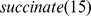 (Table 2). The numbers in bracket represent a metabolite. In 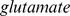 metabolism, the selected start sites for infection spread are 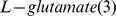 and 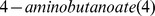 respectively. Initially, in infection-free state, 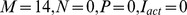 and 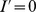. We executed our simulation algorithm by considering 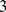 as the start site of infection initiation in the 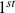 run (Fig. S1 in File S1). The front propagation of 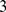, 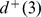 = 5 as it was connected to five metabolites, namely, 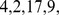 and 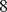. The burning probability of 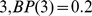, stating the fact that the chance of infection spread to the five connected metabolites was 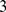, i.e., 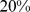 each. Similarly for metabolite 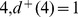, as it was connected to only one metabolite, i.e. 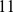, whereas the burning probability, 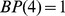, suggesting that the chance that metabolite 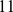 would be infected was very high (100%). Next the front propagation of 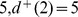, as it was connected to five metabolites, namely, 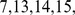 and 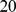 whereas its burning probability, 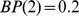, again suggesting the fact that chance of infection spread through 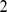 is 20%. Furthermore, for metabolites 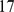 and 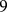, the front propagation values are 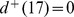 and 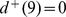, suggesting that they had no possibility of infection spread through them. For metabolite 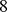, the front propagation, 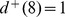, as it was connected to only one metabolite, namely, 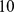 and had a burning probability, 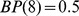, having 50% chance that infection would spread through it. Similarly for metabolite 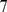, the front propagation, 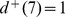, as it is connected to one metabolite, i.e. 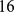, whereas its burning probability, 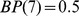. Furthermore, front propagation value of metabolite 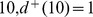, as it is connected to one metabolite, i.e., 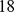 and had a burning probability, 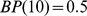. Moreover, front propagation value for other metabolites 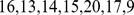 and 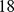 was 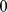 suggesting no infection spread through these metabolites (Fig. 4).

**Table 1:** Initiating metabolites for Type I *Diabetes mellitus* in *H. sapiens*.

Metabolic pathway	Infection start site	Number of connecting links	Reaction links
Glutamate metabolism	L-glutamate	05	L-glutamate→4-aminobutanoate
			L-glutamate→*ρ*_4_-L-glutanyl-L-cysteine
			L-glutamate→L-glutamyl-tRNA Glu
			L-glutamate→L-glutamine
			L-glutamate→2-oxoglutarate
	4-aminobutanoate	02	4-aminobutanoate→succinate semialdehyde
			4-aminobutanoate→L-glutamate
*β*-alanine metabolism	*β*-alanine	05	*β*-alanine→*beta*-alanyl-N-pi-methyl-L-histidine
			*β*-alanine→L-aspartate
			*β*-alanine→3-ureidopropionate
			*β*-alanine→*beta*-aminopropion aldehyde
			*β*-alanine→3-oxopropanoate
	L-aspartate	01	L-aspartate→*beta*-alanine
Taurine and hypotaurine metabolism	3-sulfino-L-alanine	02	3-sulfino-L-alanine→hypotaurine
			3-sulfino-L-alanine→L-cysteine
	Taurine	03	Taurine→taurocholate
			Taurine→L-cysteate
			Taurine→5-glutamyl-taurine
	L-cysteate	01	L-cysteate→taurine
	Hypotaurine	02	Hypotaurine→3-sulfino-L-alanine
			Hypotaurine→cysteamine
Butanoate metabolism	4-aminobutanoate	02	4-aminobutanoate→succinate semialdehyde
			4-aminobutanoate→L-glutamate
	L-glutamate	01	L-glutamate→4-aminobutanoate

**Table 2:** Reactions and metabolites involved in glutamate metabolism in *H. sapiens*.

Reactions
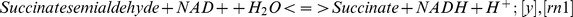
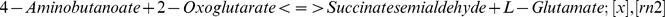
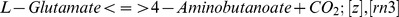
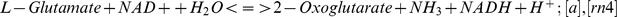
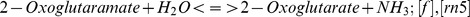
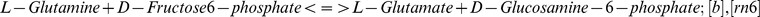

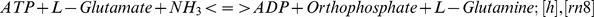
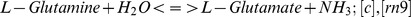
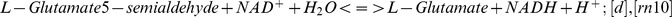
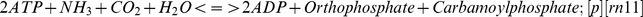
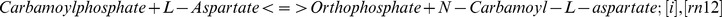

As this simulation had been done only on the basis of structural aspects of the network, biological significance needs to be associated with this model. For this purpose, we calculated certain quantitative parameters associated with it. Thus, for 
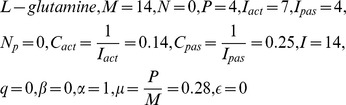
 and 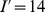. Similarly, we observed that 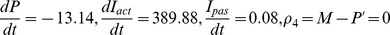. Thus, the critical factor, 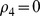, even after selecting 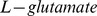 as infection start site. For the 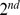 run, we selected metabolite 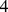 as start site for infection spread (Fig. S in File S1). It had a front propagation, 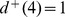, as it was connected to 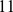, and had a burning probability 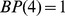, suggesting the chance of infection spread as 100%. Continuing the same strategy, we found that for metabolite 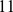, the front propagation, 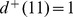, as it was connected to 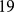. Furthermore, for 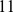 and 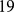, the burning probability values were 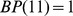 and 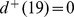 suggesting the termination of infection spread (Fig. 5). A combat mechanism also occured simultaneously along with infection spread. For instance, when 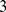 got infected, 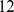 acted in accordance that results in healthy production of 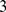. The reason being, back propagation of metabolite 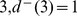 and its combating probability, 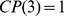, demonstrating the fact that even if it got infected combating the infection occurred. In this case, the combating edge was in between metabolites 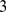 and 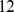 (Fig. S3 in File S1). In case of metabolite 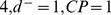 was selected as the start site for infection, 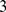 acted in accordance to combat the infection. Thus, the combating edge was in between 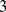 and 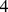 respectively (Fig. S4 in File S1). Now, for 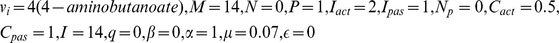 and 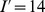. Thus, 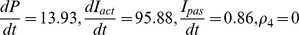. Thus, selecting 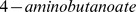 again gave 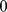 as critical factor.

In case of 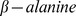 metabolism, two runs were possible as only two possible metabolites, 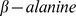 and 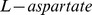 acted as possible start sites for infection. Selecting 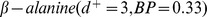 as start site results in infecting 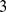 metabolites, namely, 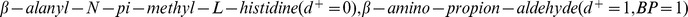, and 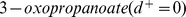. The metabolite 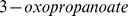 infected 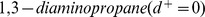 resulting in no further infection progress (Section S2 Fig. S6 in File S1). Now, for biological significance and validation purpose,we found that 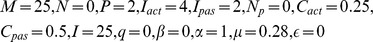 and 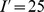. Thus, 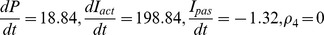. We observed that critical factor was 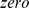 using ODE, confirming our previous analysis using graph-based study. It is also known from literature that altering the level of 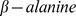 may affect the formation of 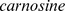 in cells resulting in several side effects such as reduction in antioxidant level as well as other carbohydrate-based disorders [38], [39]. In case of combat operation,initiating with 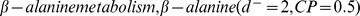 had two possible combating edges with metabolites 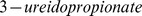 and 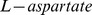 respectively (Section S2 Fig. S7 in File S1) [17]. Furthermore, L-aspartate (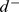 = 1, CP = 1) has only one possible combating edge with metabolite 3-ureidopropionate (Section S2 Fig. S8 in File S1). Furthermore, selecting 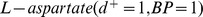 as infection spread infects only 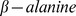, which further spreads the infection (as discussed previously) (Section S2 Fig. S9 in File S1). Also, 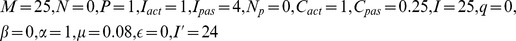. Thus, 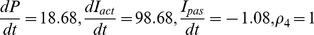, with critical factor as non-zero, thus having possibility for infection spread through metabolite. From literature, it is known that 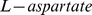 is essential for metabolic demand and is categorized as an important precursor for 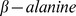, thereby significantly participating in insulin functioning [40], [41].

Furthermore, in 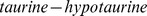 metabolism, four runs were possible as 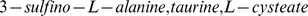 and 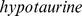 could act as start site. For the 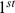 run, when 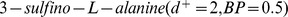 acted as the start site, it infects 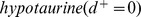 and 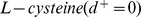 resulting in no further progress (Section S2 Fig. S10 in File S1). Here, 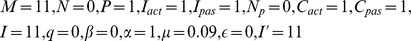. Thus, 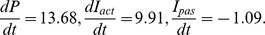 We have found that the fact 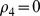 validates our result. From literature, we found that 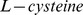 inhibits insulin release from pancreatic 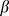-cell, thereby playing a significant role [42], [43]. This proved our previous conclusion that selecting 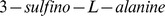 infected 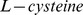 which might further effect the production of insulin [42]. Meanwhile, in 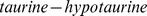 metabolism, 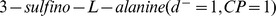 had only one possible combat edge with 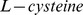. Moreover, if 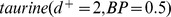 was selected as start site (for the 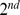 run), it infected 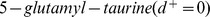 and 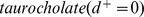 resulting in no more infection spread (Section S2 Fig. S11 in File S1) [44]. Now, 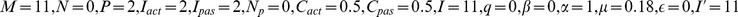. Thus, 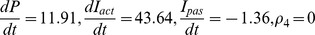. Also, 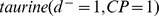 had a combat edge with 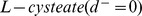 which had no further mechanism of combat (Section S2 Figs. S12–S14 in File S1) [45]. Similarly for the 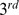 run, when 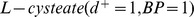 acted as start site, it infects taurine resulting in further infection spread (as discussed previously)(Section S2 Fig. S15 in File S1), for which 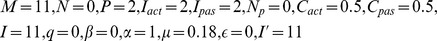. Thus, 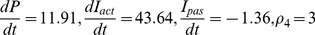. We observed that critical factor was high in this case, thus validating our result again. 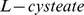 had been found to be important in synthesis of various other metabolites like 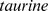, which plays an essential role in improving insulin resistance [46], [47]. As per our finding, infecting taurine may drastically spread this infection throughout. Lastly for the 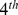 run, selecting 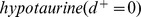 did not result in any further progress of infection (Section S2 Fig. S16 in File S1) [45]. In this case, 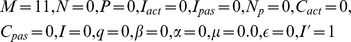. Thus, 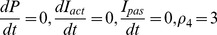 [48]. Results on Infection spread analysis in butanoate metabolism has been discussed in Section S2 Figs. S17–S20 in File S1.

### Detecting feedback reactions in metabolic pathways

Here, we discuss the results that we got by identifying the presence of feedbacks in the above mentioned four metabolic pathways. We performed the analysis in two steps. First, we identified the presence of a feedback pattern using the algorithm discussed in section ‘Methodology’, and second, we validated this property biologically using the notation previously discussed in section ‘Analyzing feedbacks’. For glutamate metabolism, we found the presence of three possible feedback links (all sequential links), in the form of reactions 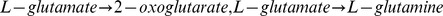 and 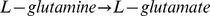 respectively (Section S3 Fig. S21a in File S1). This detection was performed on the basics of ‘Sequential Links’ algorithm in section ‘Methodology’. Thus, 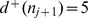 (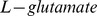), 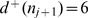 (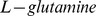) and 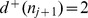 (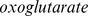). Now, for 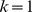, links exist between 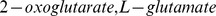 and 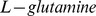 respectively. For the other feedback categories, meaningful results did not exist. For validation, we found 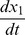 for 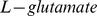 and 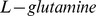, whereas 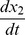 for 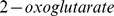 and 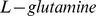. Thus, for initial concentration of 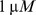 for all the metabolites, 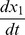 for 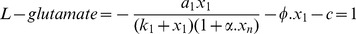 and 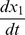 for 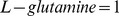, whereas 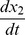 for 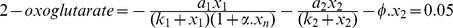 and 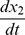 for 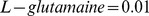 respectively [49], [50]. Thus, with time, the concentration of 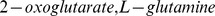 and 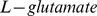 follows a negative downgrade, making the reactions behaved in a feedback manner [9], [50].

Similarly, for 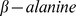 metabolism, only one reaction displayed properties of feedback, namely, 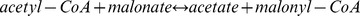, where sequential link is established. Thus, 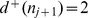 (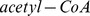). Now, for 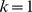, links existed between 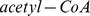 and 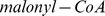. For the purpose of validation, we found that 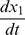 for 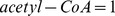 and 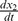 for 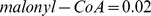. Furthermore, concentration of 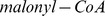 decreases with time (Section S3 Fig. S21b in File S1) [49]. In case of 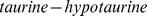 metabolism, sequential link was established in two reactions, namely, 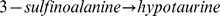 and 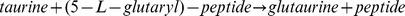. For 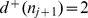 (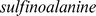) and 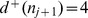 (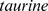) and 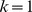, links were present between 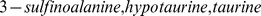 and 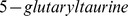 [51], [52]. Thus, for the purpose of validation, we found, 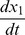 for 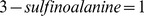, 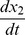 for 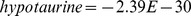, 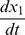 for 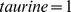, 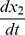 for 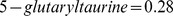, where concentration of 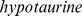 decreased with time whereas that of 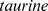 increased (Section S3 Fig. S21c in File S1) [9], [45]. Results on feedback detection in butanoate metabolism has been discussed in Section S3 Fig. S21d in File S1.

### Performing local sensitivity analysis

Every reaction model of metabolic pathways contain a number of parameters like initial concentration of metabolites and kinetic constants, whose values are not known exactly. Altering these parameters change the behavior of the model, and also specify whether the model is dependent on these parameters or not. This is extremely essential in disease networks, as a right combination of parameters can be used to analyze the dynamics of the network. Local sensitivity analysis describes how much does a specific parameter change the behavior of the model. We have calculated the sensitivity values for different parameters based upon time courses using the finite-difference method (already discussed in section ‘Methodology’). Here, we have performed sensitivity analysis using three different conditions, namely, concentration fluxes of reactions with initial concentrations, non-constant concentration of species with initial concentrations, and concentration rates with initial concentrations [27].

The sensitivities of all parameters with respect to all reactions in the model has been calculated and displayed in Table 3 where the columns correspond to the parameters (both metabolites and kinetic constants) and rows to the reactions. Let us consider the line labeled ‘(rn:R4).Flux’ (Table 3), where the numbers described how the flux of reaction ‘(rn:R4).Flux’ (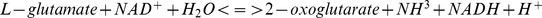) changed with concentrations of different parameters. Here, 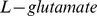 is a substrate with initial concentration 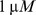 and 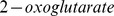, a product showing negative gradient with concentration of 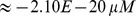, leading to a lower flux, which might ultimately lead to the decrease in reaction rate [27]. A sensitivity value equal to zero indicates that the metabolite concentration has no influence on the reaction rate. It is also important to know that sensitivity values are dominated by changes in enzyme concentrations, as they only measure the effects of changing the overall rate of reactions. Similarly, for the reaction flux, ‘(rn:R6).Flux’, 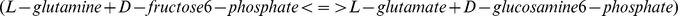, concentration of the reactant (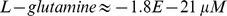) decreased, as well as for reaction flux, ‘(rn:R7).Flux’, 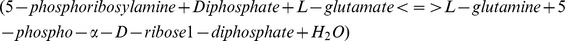, where concentration of the reactant (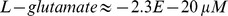) decreased too, indicating positive correlation and normal reaction rate. Furthermore, values corresponding to non-constant concentration of species with initial concentrations, negative gradients has been indicated in case of metabolites 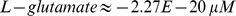, 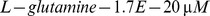 and 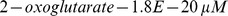 for genes 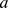, 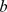 and 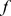 respectively. Lastly, in Table 3 concentration rates with initial concentrations did not provide any meaningful result as values were 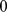 [51].

**Table 3:** Representing values corresponding to local sensitivity analysis done on glutamate metabolism; rows signify fluxes and columns denote the metabolites participating in various reactions.

Reaction ID	Flux ID (rn:)	L-glut amate	L-glut amine	4-amino butan oate	5-phos phorib osylam ine	D-gluc osami ne-6P	succ inate semial dehyde	succ inate	2-oxo gluta rate	2-oxo glutar amate	D-fruc tose 6P	5-P-*α*-D ribose 1-diP	L-glutam ate-5 semial dehyde	L-asp art ate
y	(1)	0	0	0	0	0	0.023467	1.98E-18	0	0	0	0	0	0
x	(2)	0	0	0.012564	0	0	2.03E-20	0	0.054354	0	0	0	0	0
z	(3)	0	0.015343	1.23E-21	0	0	0	0	0	0	0	0	0	0
a	(4)	0	0	0	0	0	0	0	2.10E-20	0	0	0	0	0
f	(5)	0	0	0	0	0	0	0	1.30E-21	0.067803	0	0	0	0
b	(6)	1.21E-20	1.80E-21	0	0	1.23E-21	0	0	0	0	0.189356	0	0	0
g	(7)	2.30E-20	0	0	1.18E-19	0	0	0	0	0	0	1.23E-18	0	0
h	(8)	0.185643	0	0	0	0	0	0	0	0	0	0	0	0
c	(9)	2.41E-19	1.20E-18	0	0	0	0	0	0	0	0	0	0	0
d	(10)	0	1.18E-19	0	0	0	0	0	0	0	0	0	0.198234	0
i	(11)	0	0	0	0	0	0	0	0	0	0	0	0	0.023876

Validating the above sensitivity output with the already generated fire spread result on glutamate metabolism gave us some interesting conclusions. Selecting 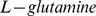 as infection initiation site led to 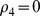 (Section S4 Table S1 in File S1). It suggested that though infection initiated at this site, all infected metabolites were ultimately cured. Furthermore, performing local sensitivity analysis indicated that concentration of infected 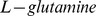 decreased with time suggesting the fact that since there was no negative flux associated with it, all infected metabolites were cured and glutamate metabolism functioning was not affected [27]. Similarly, for 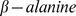 metabolism, in case of reaction flux ‘(rn:R000003).Flux’, (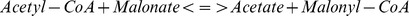) (Section S4 Table S2 in File S1), concentration of 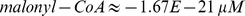 decreased, that suggested that product concentration decreased with time, signifying its possible role to act in accordance with 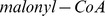. Similarly, concentration of both 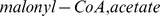 had a negative gradient values corresponding to non-constant concentration of selected metabolites [53]. Furthermore, we also observed that concentration of 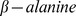, selected as infection initiation site decreased beyond 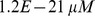. Also, concentration of 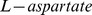 decreased beyond 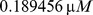 in a manner supported by common enzymatic reaction [53], [54].

Moreover, in case of 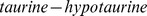 metabolism, the reaction flux ‘(rn:R02466).Flux’, (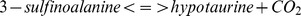) (Section S4 Table S3 in File S1) has 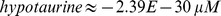 decreasing in negative gradient suggesting a possible role in inhibition. Similarly, in reaction flux, ‘(rn:R01687).Flux’, (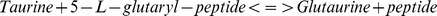) (Section S4 Table S3 in File S1), 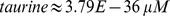 had a positive gradient. Now, in (Section S4 Table S3 in File S1), with non-constant concentration of species with respect to initial concentration displayed a negative gradient for 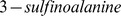 with 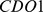 as gene. We observed that concentration of 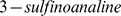 had a positive gradient even after 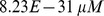 and did not give rise to non-zero critical factor [49], [55]. But, selecting 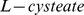 had a non-zero critical factor, which could be clearly identified using its concentration values of 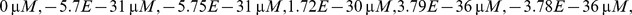 suggesting its possible role in inhibiting the reaction rate [27], [51]. In this section we looked into the metabolic pathways from a local structure point of view, which suffers from several disadvantages such as, their role to investigate the model behavior in the immediate region around the nominal parameter values and only consider changes to one parameter at a time, while all other parameters are fixed to their nominal values. Thus, to understand this situation, we performed global sensitivity analysis of infected metabolic pathways [55]. Local sensitivity analysis results on butanoate metabolism has been discussed in Section S4 Table S4 in File S1.

### Performing global sensitivity analysis

**Figure 7:**
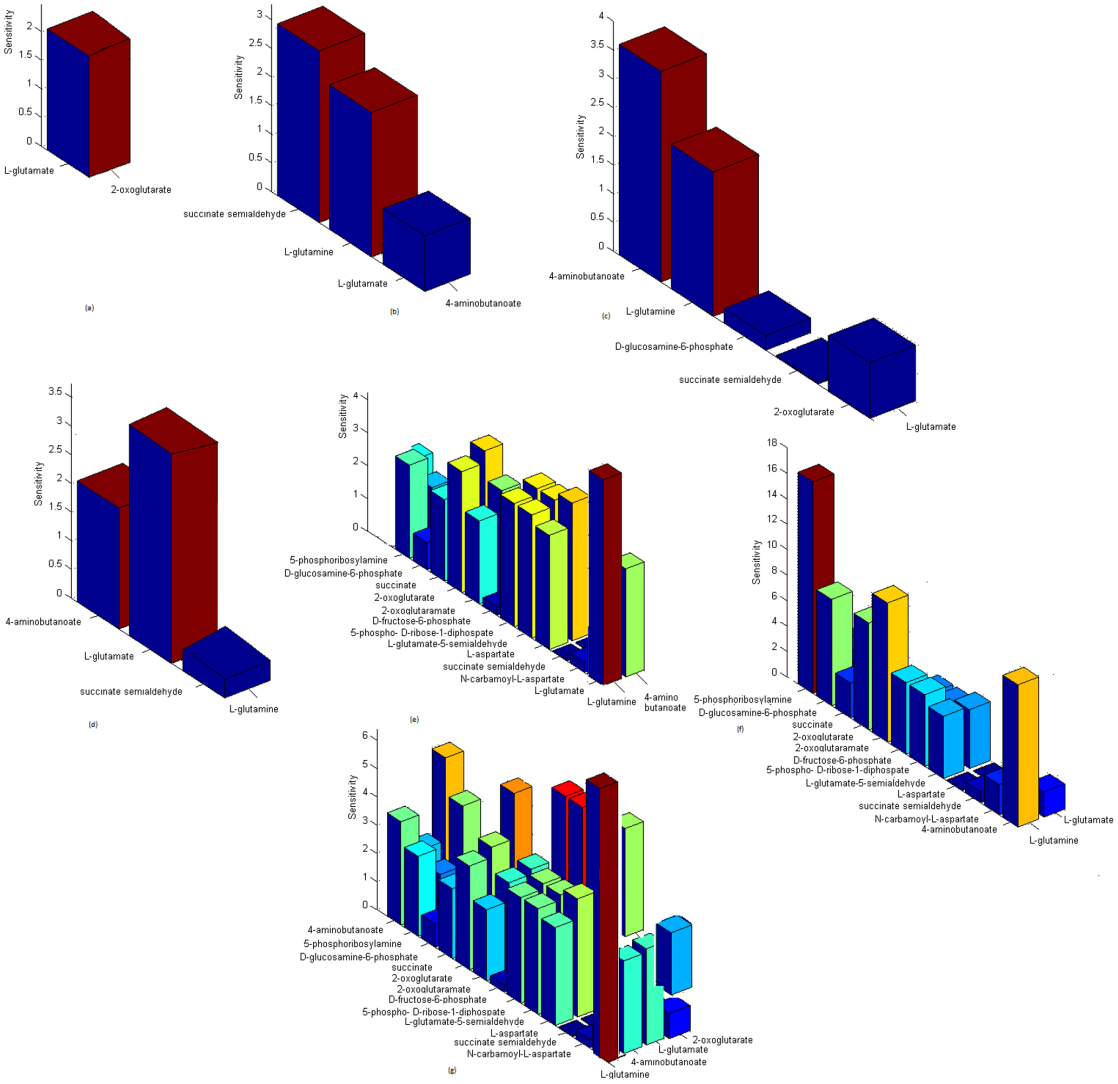
Plots representing global sensitivity analysis performed on glutamate metabolism for metabolites. (a) 2-oxoglutarate, (b) 4-aminobutanoate, (c) L-glutamate, (d) L-glutamine, (e) L-glutamine, 4-aminobutanoate (group effect), (f) L-glutamine, L-glutamate (group effect), and (g) L-glutamine, 4-aminobutanoate, L-glutamate, 2-oxoglutarate (group effect).

We have discussed previously that local sensitivity analysis considers changes to one parameter at a time, whereas in biological systems multiple parameters might act together to produce an effect. Thus, in a diseased network like T1D, it is extremely important to understand the role of multiple parameters in causing the disorder. For the same purpose, we implemented Sobol's method of global sensitivity analysis [28]. We have restricted ourselves to analyzing only those metabolites that we selected as infection start sites. We have studied the effect of metabolites upon one another, analyzing them individually as well as in groups of one, two, three and more at a time. For instance, in case of 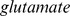 metabolism, we selected four metabolites, namely, 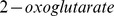, 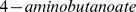, 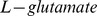 and 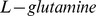. In the 1*^st^* run, we studied the effect of 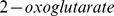 over others, 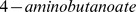 over others and so on. In the 2*^nd^* run, we studied in groups of two, like effect of 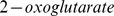 and 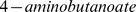 over others and so on. In the 3*^rd^* run, we studied in groups of three, whereas, in the 4*^th^* run, we studied the effect of metabolites in groups of four. Thus, we could validate our forest fire hypothesis and studied whether the effects of these metabolites as proposed in the fire spread model are true or not. Thus, for 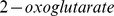, we found its maximal effect on 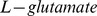, whereas for 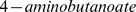, the maximal effect was found on 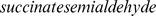, followed by 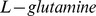 and 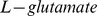. Similarly for 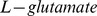, the maximal effect produced on 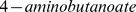, followed by 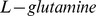 and 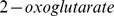, whereas for 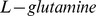 the maximal effect was on 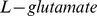 respectively (Figs. 7a–c) [56], [57].

Considering two metabolites at a time, for 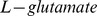 and 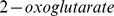, maximal group effect was on 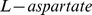, whereas for 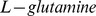 and 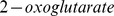 the effect was identified on 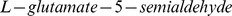, for 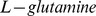 and 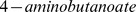 the effect was seen on 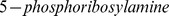, for 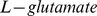 and 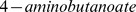 the effect was identified on 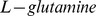, for 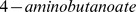 and 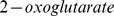 the effect was seen on 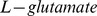. Next, considering three metabolites at a time, for 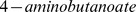, 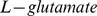 and 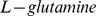 effect was seen on 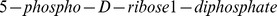, 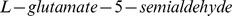, 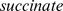 and 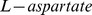 whereas for 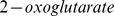, 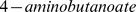, 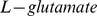 effect was seen on 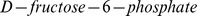 only. Lastly, considering all the four metabolites at one time, we identified the effect on 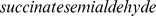, 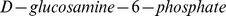, 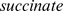, 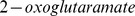 and 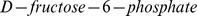 respectively. We observed that the total number of affected metabolites due to these start sites, as per fire spread model was 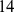, which was equivalent to the total number of affected metabolites as found in the global sensitivity plot (Figs. 7d–g) [55], [57].

For 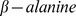 metabolism, we observed that considering 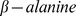 causes maximal effect on 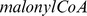, 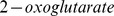, 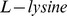, 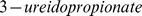 and 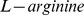, whereas for 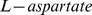 effect was seen on 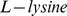, 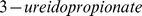 and 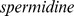. Moreover, considering both 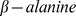 and 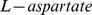, group effect was seen on 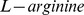, 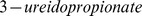 and 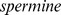, whereas for 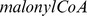 and 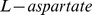, group effect was observed on 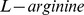, 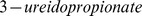 and 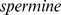. Similarly, for 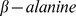 and 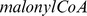, group effect was observed on 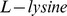, 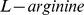 and 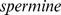. Finally, considering 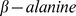, 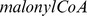 and 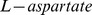, group effect was seen on 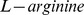, 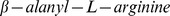 and 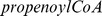 respectively. Furthermore, the fire spread model suggested an effect of over 15 metabolites due to infection spread, which was identical to what global sensitivity analysis predicts, which validated our previous finding (Section S5(SI) Figs. S22 a–f in File S1) [55], [56].

For 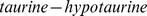 metabolism, considering 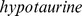 maximal effect was observed on 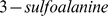, 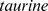, 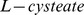, 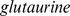, and for 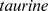, maximal effect was seen on 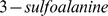, 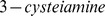, 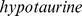, 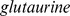, for 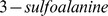 effect was seen on 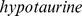, 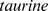, 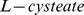 and 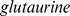. Moreover, considering both 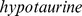 and 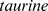, maximal group effect was seen on 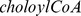, 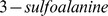, 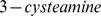, 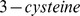 and 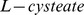, for 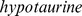 and 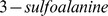 maximal group effect was seen on 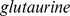, 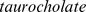, 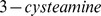, 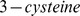 and 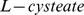, whereas for 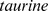 and 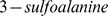 maximal effect was seen on 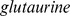, 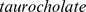, 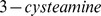, 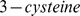 and 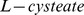 [28]. Finally, taking all three metabolites, maximal effect was seen on 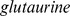, 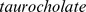, 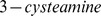, 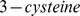, 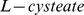 and 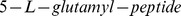. Moreover, the fire spread model suggested an effect of over 12 metabolites due to infection spread, which was similar to our results shown using global sensitivity analysis, thus validating our results (Section S5(SII) Figs. S23 a–g in File S1) [45], [55]. Global sensitivity analysis results on butanoate metabolism is discussed in Section S5(SIII) Figs. S24a–g in File S1.

### Analyzing the damage caused in metabolic pathways due to infection spread

When we considered an infected metabolite, high burning probability resulted in greater chance that it could infect the associated metabolic pathway. Similarly, if combating probability was high, then the metabolites were cured and the associated metabolic pathway was restored. We calculated a possible range of critical values for all metabolic pathways in *H. sapiens* (Fig. S5 in File S1). It illustrated the effect of infection spread and its subsequent curing. Critical value of a particular metabolic pathway suggested the overall infection scenario, giving insight into the number of infected metabolites that could not be cured after combat analysis is performed. Table S5 (in File S1) represents the list of infected, cured and uncured metabolites. From Fig. S5 (in File S1), it was evident that metabolic pathways under carbohydrate metabolism had critical values ranging from 1 to 7 having an average of 4.59, from 1 to 35 under amino acid metabolism with average of 6.22, from 1 to 36 under lipid metabolism range average of 6.89, from 1 to 4 under energy metabolism with average of 2.2 and from 1 to 8 under co-factors and vitamins metabolism with an average of 2.8 respectively (Section S6(SI–SII) Tables S5–S6 in File S1). Thus, critical value ranging from 1–5 were less prone to infection, 5–10 were more prone to infection, whereas value greater than 10 were most prone to infection [58].

This was because critical value depends on the number of metabolites cured with respect to metabolites infected [59]. A higher critical value indicated that less number of infected metabolites were cured, whereas a less critical value indicated more number of infected metabolites cured. If critical value is 0, then all infected metabolites were cured. In case of glutamate metabolism critical value was 0, when site of infection was both L-glutamate and 4-aminobutanoate. This signifies the fact that all the metabolites that became infected due to selection of either L-glutamate or 4-aminobutanoate were cured. In case of 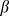-alanine metabolism, critical value was 0 when start site of infection was 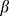-alanine, whereas it was 1 when infection start site was L-aspartate, indicating that L-aspartate remained infected even after the combat process was over [60]. In case of taurine and hypotaurine metabolism critical value was observed to be 0 (infection start site = 3-sulfino-L-alanine and taurine), whereas it was 4 when infection start site was L-cysteate. For the later case, reason for high critical value was due to the unsuccessful combat mechanismn, in which taurine, L-cysteate, taurocholate and 5-glutamyl taurine remained infected. Furthermore, in butanoate metabolism critical value was 0 when infection start site was 4-aminobutanoate, whereas it was 4 when infection start site was L-glutamate as the 4-aminobutanoate, succinate semialdehyde, succinate and L-glutamate remain infected even after combat process ends. The critical values could be further used to analyze the degree of fitness of a metabolic pathway in case of infection spread, and could be further utilized to study the robustness of the metabolites involved in a metabolic pathway. Furthermore, we have also performed various perturbations on the given metabolic pathways (as discussed in Section ‘Modeling metabolic pathways quantitatively’). The various perturbation values for those metabolic pathways are shown in Tables S7–S10 (Section S7 in File S1).

## Discussion

This work facilitated the study of metabolic networks and simulate the infection caused in a healthy network with implementation in certain pathways involved in Type I *Diabetes mellitus* in *H. sapiens*. The aim of this study was to evaluate whether each metabolite is infected by any chance, and the nature as well as extent of this infection. Moreover, we have also studied whether this infection spread could be combated as well as the infected metabolites could be cured. We also identified the extent of infection by calculating the critical value using both burning as well as combating probability. This simulation model considered a metabolite which was susceptible to infection via an infected metabolite. Once a metabolite was infected, it spread the infection, which harmed the network but also started recovering if there was a regulation provided to the metabolite. Also, there was a chance that this cured metabolite was again susceptible to the infection spread.

We implemented this method in four metabolic pathways for *H. sapiens* involved in Type I *Diabetes mellitus*, namely, glutamate metabolism, 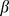-alanine metabolism, taurine and hypotaurine metabolism and butanoate metabolism. The reason for selecting these metabolic pathways was due to the involvement of two important genes GAD and INS that have major role in Type I *Diabetes mellitus*. The number of start site of infection spread for these four metabolic pathways were found to be 10, namely, L-glutamate, 4-aminobutanoate (for glutamate metabolism), 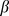-alanine, L-aspartate (for 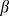-alanine metabolism), 3-sulfino-L-alanine, taurine, L-cysteate, hypotaurine (for taurine and hypotaurine metabolism), and 4-aminobutanoate, L-glutamate (for butanoate metabolism). Furthermore, for tracking the path of infection spread through these infection start sites as well as identifying their containment strategy, we found the burning probability and combating probability values as 0.2 and 1 (L-glutamate), 1 and 1 (4-aminobutanoate), 0.33 and 0.5 (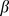-alanine), 1 and 1 (L-aspartate), 0.5 and 1 (3-sulfino-L-alanine), 0.5 and 1 (taurine), 1 and 0 (L-cysteate), 0 and 0 (hypotaurine), 1 and 1 (4-aminobutanoate), 1 and 0 (L-glutamate) respectively. Thus, out of these 10 probable start site for infection spread L-cysteate, hypotaurine and L-glutamate have no ability to combat the infection spread, whereas the other metabolites have the combating ability ranging from 33% to 100%. These ten probable infection start sites may be targeted to explore the effects of long-term infection combat and cure.

For implementing the fire spread, we used strategies, based on quantitative studies and graphs. The quantitative strategy using ODEs implements the fire spread using mathematical models and expressions. For biological validation, we used the sensitivity analysis for identifying the nature and property of these metabolites and their role in disease spread. In our model we do not consider any metabolite to become immunized to the infection spread and consider them equally susceptible to other infected metabolites against infection spread. One of the effective approaches in this case is curing the infected metabolites and vaccinating the uneffected ones with a probability proportional to their conductivities, so that a greater proportion of metabolites of high connectivity are vaccinated than metabolites with low connectivity. Another strategy is specifically targeting the hub metabolites by vaccinating all metabolites in the pathway of connectivity higher than some threshold value. The processes of infection and curing run for a specific number of iterations, depending on the number of metabolites in the metabolic pathway. We have assigned a maximum iteration value of 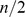, where 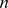 is the total number of metabolites in the metabolic pathway. The reason for this threshold is that after the iteration value is 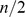, the results converge and there is no further need to continue performing further iterations. After the infection is combated and the number of iterations is complete, the critical value, signifying the number of metabolites that cannot be cured, is calculated.

From our analysis, we have also found that in *H. sapiens* metabolic pathways under carbohydrate metabolism have a range of critical values from 1 to 7, under amino acid metabolism from 1 to 35, under lipid metabolism from 1 to 36, under energy metabolism from 1 to 4 and under metabolism of co-factors and vitamins from 1 to 8. Furthermore, from this study we want to mention that critical values ranging from 1–5 is less prone to infection, 5–10 is more prone to infection, whereas value greater than 10 are most prone to infection. We would also like to make a note on some recent advances in systems biology approaches, such as flux balance analysis, which have been successful in idenitfying optimal metabolic pathways and extreme pathways. But, the volume of work that have been done in correlating sensitivities, both local and global, with FBA, as well as judging the system states of a network is less. Furthermore, less work have been performed in areas of detecting and quantifying ‘feedback’ using certain conventional techniques like FBA. Thus, novelty of our approach lies in that we have correlated system state identification, feedback detection, as well as sensitivities studies in diseased state pathways. This investigation can be taken one step further by analyzing the density factor as well as applying time constraints to the infection caused in the metabolic networks. Finally, we can even extend this method to analyze the patterns associated with epidemiological and endemic networks.

## Supporting Information

File S1
**Supporting information.** Figure S1. Spread of infection in glutamate metabolism with infection start site as ‘3’. Figure S2. Spread of infection in glutamate metabolism with infection start site as ‘4’. Figure S3. Combat process in glutamate metabolism with infection start site as ‘3’. Figure S4. Combat process in glutamate metabolism in *H. sapiens* with infection start site as ‘4’. Figure S5. Plot representing distribution of critical values in all metabolic pathways in *H. sapiens*. Figure S6. 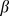-alanine metabolism; Infection start site = 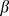-alanine. Figure S7. 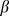-alanine metabolism; Infection start site = L-aspartate. Figure S8. 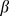-alanine metabolism; Combat analysis for infection start site = 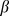-alanine. Figure S9. 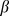-alanine metabolism; Combat analysis for infection start site = L-aspartate. Figure S10. Taurine and hypotaurine metabolism; Infection start site = 3-sulfino-L-alanine. Figure S11. Taurine and hypotaurine metabolism; Infection start site = taurine. Figure S12. Taurine and hypotaurine metabolism; Combat analysis for infection start site = 3-sulfino-L-alanine. Figure S13. Taurine and hypotaurine metabolism; Combat analysis for infection start site = taurine. Figure S14. Taurine and hypotaurine metabolism; Combat analysis for infection start site = L-cysteate. Figure S15. Taurine and hypotaurine metabolism; Infection start site = L-cysteate. Figure S16. Taurine and hypotaurine metabolism; Infection start site = hypotaurine. Figure S17. Butanoate metabolism; Infection start site = 4-aminobutanoate. Figure S18. Butanoate metabolism; Infection start site = L-glutamate. Figure S19. Butanoate metabolism; Combat analysis for infection start site = 4-aminobutanoate. Figure S20. Butanoate metabolism; Combat analysis for infection start site = L-glutamate. Figure S21. Feedback analysis. Figure S22. Global sensitivity analysis of 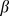-alanine metabolism. Figure S23. Global sensitivity analysis of taurine-hypotaurine metabolism. Figure S24. Global sensitivity analysis of butanoate metabolism. Table S1. Target function, non-constant concentration of species, variable, initial concentration in glutamate metabolism. Table S2. Target function, concentration fluxes of reactions, variable, initial concentration in 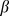-alanine metabolism. Table S3. Target function, concentration fluxes of reactions, variable, initial concentration in taurine and hypotaurine metabolism. Table S4. Target function, concentration fluxes of reactions, variable, initial concentration in butanoate metabolism. Table S5. Identification of infected, cured and un-cured metabolites in the four metabolic pathways. Table S6. Critical value analysis in the four metabolic pathways. Table S7. Perturbations in 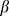-alanine metabolism. Table S8. Perturbations in taurine and hypotaurine metabolism. Table S9. Perturbations in butanoate metabolism. Table S10. Perturbations in glutamate metabolism.(DOC)
