# Supplementary material for: Simulating an Infection Growth Model in Certain Healthy Metabolic Pathways of Homo sapiens for Highlighting Their Role in Type I Diabetes mellitus Using Fire-Spread Strategy, Feedbacks and Sensitivities
Source: PLoS One. 2013 Sep 9;8(9):e69724. doi: 10.1371/journal.pone.0069724 (PMC3767837; doi:10.1371/journal.pone.0069724)
Supplement: File S1 — Supporting information. Figure S1. Spread of infection in glutamate metabolism with infection start site as ‘3’. Figure S2. Spread of infection in glutamate metabolism with infection start site as ‘4’. Figure S3. Combat process in glutamate metabolism with infection start site as ‘3’. Figure S4. Combat process in glutamate metabolism in H. sapiens with infection start site as ‘4’. Figure S5. Plot representing distribution of critical values in all metabolic pathways in H. sapiens. Figure S6. -alanine metabolism; Infection start site = -alanine. Figure S7. -alanine metabolism; Infection start site = L-aspartate. Figure S8. -alanine metabolism; Combat analysis for infection start site = -alanine. Figure S9. -alanine metabolism; Combat analysis for infection start site = L-aspartate. Figure S10. Taurine and hypotaurine metabolism; Infection start site = 3-sulfino-L-alanine. Figure S11. Taurine and hypotaurine metabolism; Infection start site = taurine. Figure S12. Taurine and hypotaurine metabolism; Combat analysis for infection start site = 3-sulfino-L-alanine. Figure S13. Taurine and hypotaurine metabolism; Combat analysis for infection start site = taurine. Figure S14. Taurine and hypotaurine metabolism; Combat analysis for infection start site = L-cysteate. Figure S15. Taurine and hypotaurine metabolism; Infection start site = L-cysteate. Figure S16. Taurine and hypotaurine metabolism; Infection start site = hypotaurine. Figure S17. Butanoate metabolism; Infection start site = 4-aminobutanoate. Figure S18. Butanoate metabolism; Infection start site = L-glutamate. Figure S19. Butanoate metabolism; Combat analysis for infection start site = 4-aminobutanoate. Figure S20. Butanoate metabolism; Combat analysis for infection start site = L-glutamate. Figure S21. Feedback analysis. Figure S22. Global sensitivity analysis of -alanine metabolism. Figure S23. Global sensitivity analysis of taurine-hypotaurine metabolism. Figure S24. Global sensitivity analysis of butanoate m [file pone.0069724.s001.doc]

**Supplementary Information**

**Contents:**

1. Section S1: Ordinary Differential Equation representations: Pages 2-10
2. Section S2: Infection spread and combat analysis: Figs. S1-S20, Pages 10-20
3. Section S3: Feedback analysis: Fig. S21, Page 21
4. Section S4: Local sensitivity analysis: Tables S1-S4, Pages 22-24
5. Section S5: Global sensitivity analysis: Figs. S22-S24, Pages 25-28
6. Section S6: Critical value analysis: Tables S5-S6, Pages 29-30
7. Section S7: Validation using step changes in input dataset: Table S7-S10, Pages 31-32

**Section A**

(SI)

β-alanine metabolism

Malonyl-CoA <=> Acetyl-CoA + CO2; [a]

L-Aspartate <=> beta-Alanine + CO2; [b]

Acetyl-CoA + Malonate <=> Acetate + Malonyl-CoA; [c]

3-Ureidopropionate + H2O <=> beta-Alanine + CO2 + NH3; [d]

beta-Alanine + 2-Oxoglutarate <=> 3-Oxopropanoate + L-Glutamate; [e]

ATP + L-Lysine + beta-Alanine <=> AMP + Diphosphate + beta-Alanyl-L-lysine; [f]

beta-Alanyl-L-lysine + H2O <=> beta-Alanine + L-Lysine; [g]

ATP + L-Arginine + beta-Alanine <=> AMP + Diphosphate + beta-Alanyl-L-arginine; [h]

5,6-Dihydrouracil + NAD+ <=> Uracil + NADH + H+; [i]

5,6-Dihydrouracil + NADP+ <=> Uracil + NADPH + H+; [j]

ATP + L-Histidine + beta-Alanine <=> AMP + Diphosphate + Carnosine; [k]

Carnosine + H2O <=> beta-Alanine + L-Histidine; [l]

5,6-Dihydrouracil + H2O <=> 3-Ureidopropionate; [m]

S-Adenosylmethioninamine + Spermidine <=> 5'-Methylthioadenosine + Spermine; [n]

3-Hydroxypropionyl-CoA <=> Propenoyl-CoA + H2O; [o]

3-Hydroxypropanoate + CoA <=> 3-Hydroxypropionyl-CoA + H2O; [p]

beta-Alanyl-L-arginine + H2O <=> beta-Alanine + Amino acid(Arg-); [q]

= - $V_{def}.\left(\frac{1.[a].[malonyl-CoA].V_{def}}{1.1+[malonyl-CoA].V_{def}-[acetyl-CoA].V_{def}}\right)$

+

= - $V_{def}.\left(\frac{1.[c].[acetyl-CoA].V_{def}.[malonate].V_{def}}{1.1+[acetyl-CoA].V_{def}.[malonate].V_{def}-[acetate].V_{def}.[malonyl-CoA].V_{def}}\right)$

+

= - $V_{def}.\left(\frac{1.[b].[L-aspartate].V_{def}}{1.1+[L-aspartate].V_{def}-[\beta-alanine].V_{def}}\right)$

= - $V_{def}.\left(\frac{1.[e].[\beta-alanine].V_{def}.[2-oxo-glutarate].V_{def}}{1.1+[\beta-alanine].V_{def}.[2-oxo-glutarate].V_{def}-[2-oxoglutarate].V_{def}.[3-oxopropanoate].V_{def}}\right)$

-

-

-

+

+

+

+

+

= - $V_{def}.\left(\frac{1.[c].[malonate].V_{def}.[acetyl-CoA].V_{def}}{1.1+[malonate].V_{def}.[acetyl-CoA].V_{def}-[acetate].V_{def}.[malonyl-CoA].V_{def}}\right)$

= + $V_{def}.\left([c].\frac{1.[acetyl-CoA].V_{def}.[malonate].V_{def}}{1.[acetyl-CoA].V_{def}.[malonate].V_{def}-[malonyl-CoA].V_{def}}\right)$

= - $V_{def}.\left(\frac{1.[d].[3-ureidopropionate].V_{def}}{1.1+[3-ureidopropionate].V_{def}-[\beta-alanine].V_{def}}\right)$

+

= - $V_{def}.\left(\frac{1.[e].[2-oxoglutarate].V_{def}.[\beta-alanine].V_{def}}{1.1+[2-oxoglutarate].V_{def}.[\beta-alanine].V_{def}-[3-oxopropionate].V_{def}.[L-glutamate].V_{def}}\right)$

= + $V_{def}.\left([e].\frac{1.[\beta-alanine].V_{def}.[2-oxoglutarate].V_{def}}{1.[\beta-alanine].V_{def}.[2-oxoglutarate].V_{def}-[L-glutamate].V_{def}}\right)$

= + $V_{def}.\left([e].\frac{1.[\beta-alanine].V_{def}.[2-oxoglutarate].V_{def}}{1.[\beta-alanine].V_{def}.[2-oxoglutarate].V_{def}-[3-oxopropanoate].V_{def}}\right)$

= - $V_{def}.\left(\frac{1.[f].[L-ly\sin e].V_{def}.[\beta-alanine].V_{def}}{1.1+[L-ly\sin e].V_{def}.[\beta-alanine].V_{def}-[\beta-alanyl-L-ly\sin e].V_{def}}\right)$

+

= - $V_{def}.\left(\frac{1.[g].[\beta-alanyl-L-ly\sin e].V_{def}}{1.1+[\beta-alanyl-L-ly\sin e].V_{def}-[L-ly\sin e].V_{def}.[\beta-alanine].V_{def}}\right)$

+

= - $V_{def}.\left(\frac{1.[h].[L-\arg inine].V_{def}.[\beta-alanine].V_{def}}{1.1+[L-\arg inine].V_{def}.[\beta-alanine].V_{def}-[\beta-alanyl-L-ly\sin e].V_{def}}\right)$

= - $V_{def}.\left(\frac{1.[q].[\beta-alanyl-L-\arg inine].V_{def}}{1.1+[\beta-alanyl-L-\arg inine].V_{def}-[\beta-alanine].V_{def}}\right)$

+

= - $V_{def}.\left(\frac{1.[i].[5,6-dihydrouracil].V_{def}}{1.1+[5,6-dihydrouracil].V_{def}-[uracil].V_{def}}\right)$

-

-

= + $V_{def}.\left([i].\frac{1.[5,6-dihydrouracil].V_{def}}{1+[5,6-dihydrouracil].V_{def}}\right)$

+

= + $V_{def}.\left([l].\frac{1.[carno\sin e].V_{def}}{1+[carno\sin e].V_{def}-[\beta-alanine].V_{def}}\right)$

-

= + $V_{def}.\left([k].\frac{1.[L-histidine].V_{def}.[\beta-alanine].V_{def}}{1+[L-histidine].V_{def}-[\beta-alanine].V_{def}}\right)$

-

= -

= -

= + $V_{def}.\left([n].\frac{1.[5-adenosyl-methionia\min e].V_{def}.[spermidine].V_{def}}{1+[5-adenosyl-methionia\min e].V_{def}.[spermidine].V_{def}}\right)$

= +

= + $V_{def}.\left([p].\frac{1.[3-hydroxypropanoate].V_{def}}{1+[3-hydroxypropanoate].V_{def}}\right)$

-

= + $V_{def}.\left([o].\frac{1.[3-hydroxypropionyl-CoA].V_{def}}{1+[3-hydroxypropionyl-CoA].V_{def}}\right)$

= - $V_{def}.\left(\frac{1.[p].[3-hydroxypropanoate].V_{def}}{1.1+[3-hydroxypropanoate].V_{def}.-[3-hydroxypropionyl-CoA].V_{def}}\right)$

(SII)

Taurine and hypotaurine metabolism

L-cysteate taurine + CO_2_; [a]

L-cysteine + O_2_ 3-sulfinoalanine; [b]

Cysteamine + O_2_ hypotaurine; [c]

3-sulfinoalanine hypotaurine + CO_2_; [a]

taurine + (5-L-glutamyl)-peptide glutaurine + peptide; [e]

taurine + choloyl-CoA taurocholate + CoA; [d]

= + $V_{def}.\left([b].\frac{1.[L-cysteine].V_{def}}{1+[L-cysteine].V_{def}}\right)$

-

= + $V_{def}.\left([c].\frac{1.[cystea\min e].V_{def}}{1+[cystea\min e].V_{def}}\right)$

+

= - $V_{def}.\left(\frac{1.[c].[3-cystea\min e].V_{def}}{1.1+[3-cystea\min e].V_{def}}\right)$

= - $V_{def}.\left(\frac{1.[b].[3-cysteine].V_{def}}{1.1+[3-cysteine].V_{def}}\right)$

= + $V_{def}.\left([a].\frac{1.[L-cysteate].V_{def}}{1+[L-cysteate].V_{def}}\right)$

-

-

= + $V_{def}.\left([a].\frac{1.[L-cysteate].V_{def}}{1+[L-cysteate].V_{def}}\right)$

= + $V_{def}.\left([e].\frac{1.[5-L-glutamyl-peptide].V_{def}.[taurine].V_{def}}{1+[5-L-glutamyl-peptide].V_{def}.[taurine].V_{def}}\right)$

= + $V_{def}.\left([d].\frac{1.[choloyl-CoA].V_{def}.[taurine].V_{def}}{1+[choloyl-CoA].V_{def}.[taurine].V_{def}}\right)$

= - $V_{def}.\left(\frac{1.[e].[5-L-glutamyl-peptide].V_{def}.[taurine].V_{def}}{1.1+[5-L-glutamyl-peptide].V_{def}.[taurine].V_{def}}\right)$

= - $V_{def}.\left(\frac{1.[d].[choloyl-CoA].V_{def}.[taurine].V_{def}}{1.1+[choloyl-CoA].V_{def}.[taurine].V_{def}}\right)$

= - $V_{def}.\left(\frac{1.[d].[choloyl-CoA].V_{def}.[taurine].V_{def}}{1.1+[choloyl-CoA].V_{def}.[taurine].V_{def}}\right)$

(SIII)

Butanoate metabolism

Pyruvate + Thiamin diphosphate <=> 2-(alpha-Hydroxyethyl)thiamine diphosphate + CO2; [a]

2 Acetyl-CoA <=> CoA + Acetoacetyl-CoA; [b]

L-Glutamate <=> 4-Aminobutanoate + CO2; [c]

Succinyl-CoA + Acetoacetate <=> Succinate + Acetoacetyl-CoA; [d]

Succinate semialdehyde + NAD+ + H2O <=> Succinate + NADH + H+; [e]

Butanoyl-CoA + NAD+ <=> Crotonoyl-CoA + NADH + H+; [f]

ATP + Butanoic acid + CoA <=> AMP + Diphosphate + Butanoyl-CoA; [g]

ATP + Acetoacetate + CoA <=> AMP + Diphosphate + Acetoacetyl-CoA; [h]

(S)-3-Hydroxy-3-methylglutaryl-CoA <=> Acetyl-CoA + Acetoacetate; [i]

(R)-3-Hydroxybutanoate + NAD+ <=> Acetoacetate + NADH + H+; [j]

4-Aminobutanoate + 2-Oxoglutarate <=> Succinate semialdehyde + L-Glutamate; [k]

(S)-3-Hydroxybutanoyl-CoA + NAD+ <=> Acetoacetyl-CoA + NADH + H+; [l]

(S)-3-Hydroxy-3-methylglutaryl-CoA + CoA <=> Acetyl-CoA + H2O + Acetoacetyl-CoA; [m]

(S)-3-Hydroxybutanoyl-CoA <=> Crotonoyl-CoA + H2O; [n]

2-Hydroxyglutarate + FAD <=> 2-Oxoglutarate + FADH2; [o]

= - $V_{def}.\left(\frac{1.[a].[pyruvate].V_{def}.[thia\min e-diphosphate].V_{def}}{1.1+[pyruvate].V_{def}.[thia\min e-diphosphate].V_{def}-[2-\alpha-hydroxy-ethyl-thia\min e-diphosphate].V_{def}}\right)$

= - $V_{def}.\left(\frac{1.[a].[thia\min e-diphosphate].V_{def}.[pyruvate].V_{def}}{1.1+[thia\min e-diphosphate].V_{def}.[pyruvate].V_{def}-[2-\alpha-hydroxy-ethyl-thia\min e-diphosphate].V_{def}}\right)$

= + $V_{def}.\left([a].\frac{1.[pyruvate].V_{def}.[thia\min e-diphosphate].V_{def}}{1+[pyruvate].V_{def}.[thia\min e-diphosphate].V_{def}}\right)$

= + $V_{def}.\left([i].\frac{1.[(S)-3-hydroxy-3-methy\lg lutaryl-CoA].V_{def}}{1+[(S)-3-hydroxy-3-methy\lg lutaryl-CoA].V_{def}-[acetoacetate].V_{def}}\right)$

+

-

= + $V_{def}.\left([b].\frac{1.[acetyl-CoA].V_{def}}{1+[acetyl-CoA].V_{def}-[CoA].V_{def}}\right)$

+

+

+

+

= + $V_{def}.\left([k].\frac{1.[4-a\min obu\tan oate].V_{def}.[2-oxoglutarate].V_{def}}{1+[4-a\min obu\tan oate].V_{def}.[2-oxoglutarate].V_{def}-[succinate-semialdehyde].V_{def}}\right)$

-

= + $V_{def}.\left([c].\frac{1.[L-glutamate].V_{def}}{1+[L-glutamate].V_{def}}\right)$

-

= - $V_{def}.\left(\frac{1.[d].[succinyl-CoA].V_{def}.[acetoacetate].V_{def}}{1.1+[succinyl-CoA].V_{def}.[acetoacetate].V_{def}.[succinate].V_{def}.[acetoacetyl-CoA].V_{def}}\right)$

= + $V_{def}.\left([i].\frac{1.[(S)-3-hydroxy-3-methyl-glutaryl-CoA].V_{def}}{1+[(S)-3-hydroxy-3-methyl-glutaryl-CoA].V_{def}-[acetyl-CoA].V_{def}}\right)$

+

-

-

= + $V_{def}.\left([d].\frac{1.[succinyl-CoA].V_{def}.[acetoacetate].V_{def}}{1+[succinyl-CoA].V_{def}.[acetoacetate].V_{def}-[acetoacetyl-CoA].V_{def}}\right)$

+

= + $V_{def}.\left([k].\frac{1.[4-a\min obu\tan oate].V_{def}.[2-oxoglutarate].V_{def}}{1+[4-a\min obu\tan oate].V_{def}.[2-oxoglutarate].V_{def}-[L-glutamate].V_{def}}\right)$

-

= + $V_{def}.\left([g].\frac{1.[bu\tan oic-acid].V_{def}.[CoA].V_{def}}{1+[bu\tan oic-acid].V_{def}.[CoA].V_{def}}\right)$

-

= + $V_{def}.\left([f].\frac{1.[bu\tan oyl-CoA].V_{def}}{1+[bu\tan oyl-CoA].V_{def}-[crotonyl-CoA].V_{def}}\right)$

+

= - $V_{def}.\left(\frac{1.[g].[bu\tan oic-acid].V_{def}}{1.1+[bu\tan oic-acid].V_{def}.[bu\tan oyl-CoA].V_{def}}\right)$

= - $V_{def}.\left(\frac{1.[i].[(S)-3-hydroxy-3-methyl-glutaryl-CoA].V_{def}}{1.1+[(S)-3-hydroxy-3-methyl-glutaryl-CoA].V_{def}.[acetoacetyl-CoA].V_{def}.[acetoacetate].V_{def}}\right)$

-

= - $V_{def}.\left(\frac{1.[j].[(R)-3-hydroxy-bu\tan oate].V_{def}}{1.1+[(R)-3-hydroxy-bu\tan oate].V_{def}.[acetoacetate].V_{def}}\right)$

= + $V_{def}.\left([o].\frac{1.[2-hydroxy-glutarate].V_{def}}{1+[2-hydroxy-glutarate].V_{def}}\right)$

-

= - $V_{def}.\left(\frac{1.[l].[(R)-3-hydroxy-bu\tan oyl-CoA].V_{def}}{1.1+[(R)-3-hydroxy-bu\tan oyl-CoA].V_{def}.[acetoacetyl-CoA].V_{def}}\right)$

-

= - $V_{def}.\left(\frac{1.[o].[2-hydroxy-glutarate].V_{def}}{1.1+[2-hydroxy-glutarate].V_{def}.[2-oxoglutarate].V_{def}}\right)$

**Section S2**

Fig. S1: Spread of infection in glutamate metabolism with infection start site as ‘3’

Fig. S2: Spread of infection in glutamate metabolism with infection start site as ‘4’

Fig. S3: Combat process in glutamate metabolism with infection start site as `3'

Fig. S4: Combat process in glutamate metabolism in H. sapiens with infection start site as `4'

Fig. S5: Plot representing distribution of critical values in all metabolic pathways in *H. sapiens*


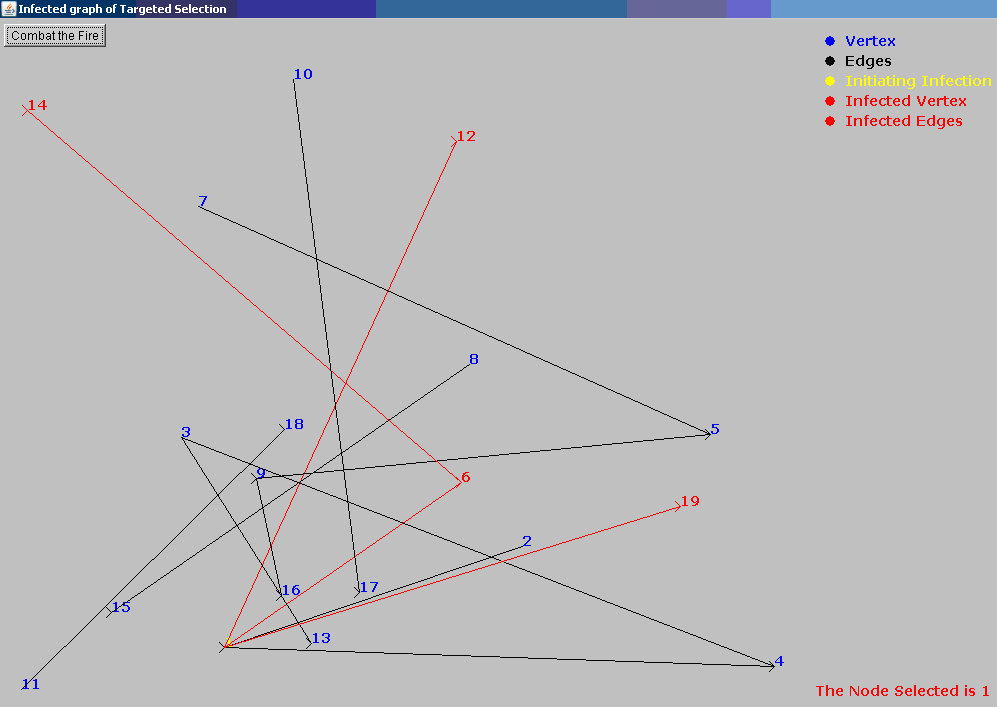


Fig. S6: β-alanine metabolism; Infection start site = β-alanine


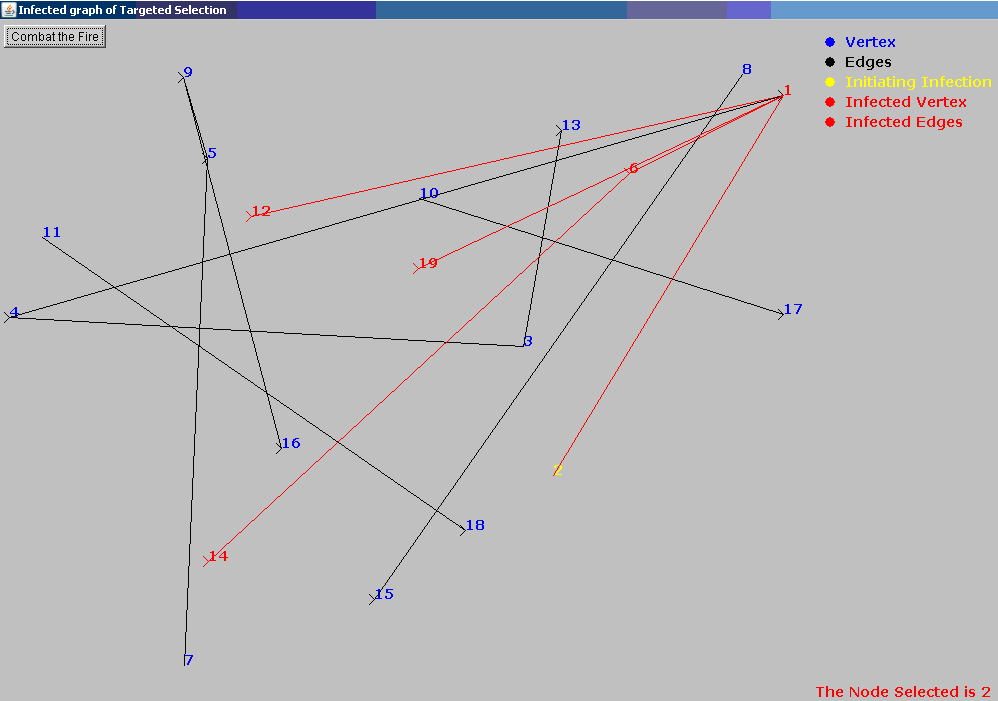


Fig. S7: β-alanine metabolism; Infection start site = L-aspartate


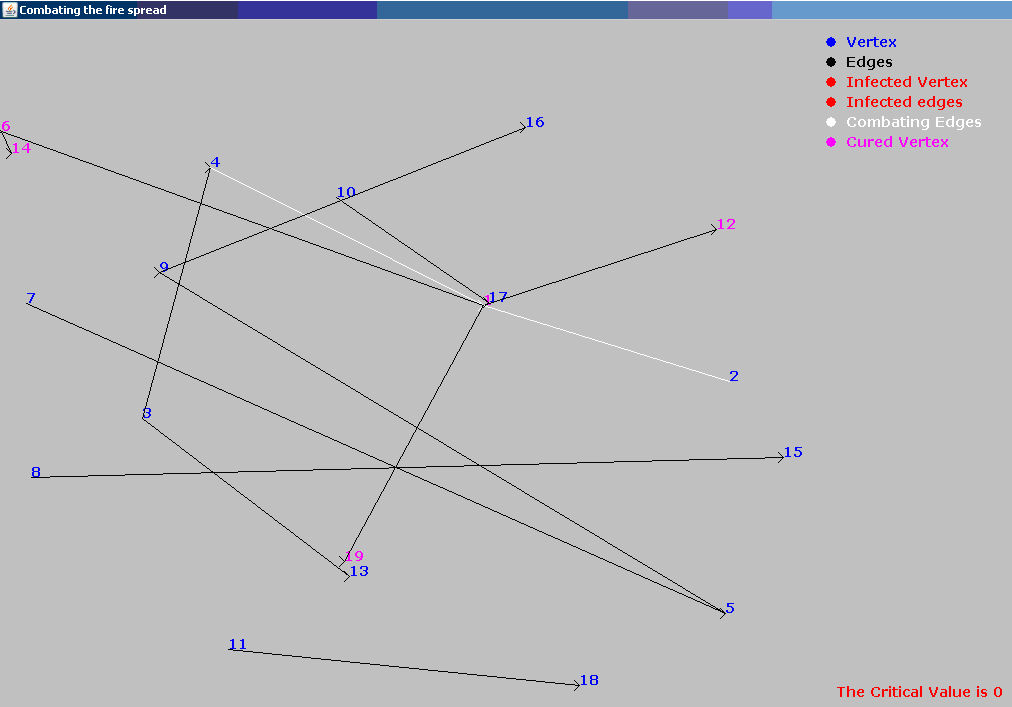


Fig. S8: β-alanine metabolism; Combat analysis for infection start site = β-alanine


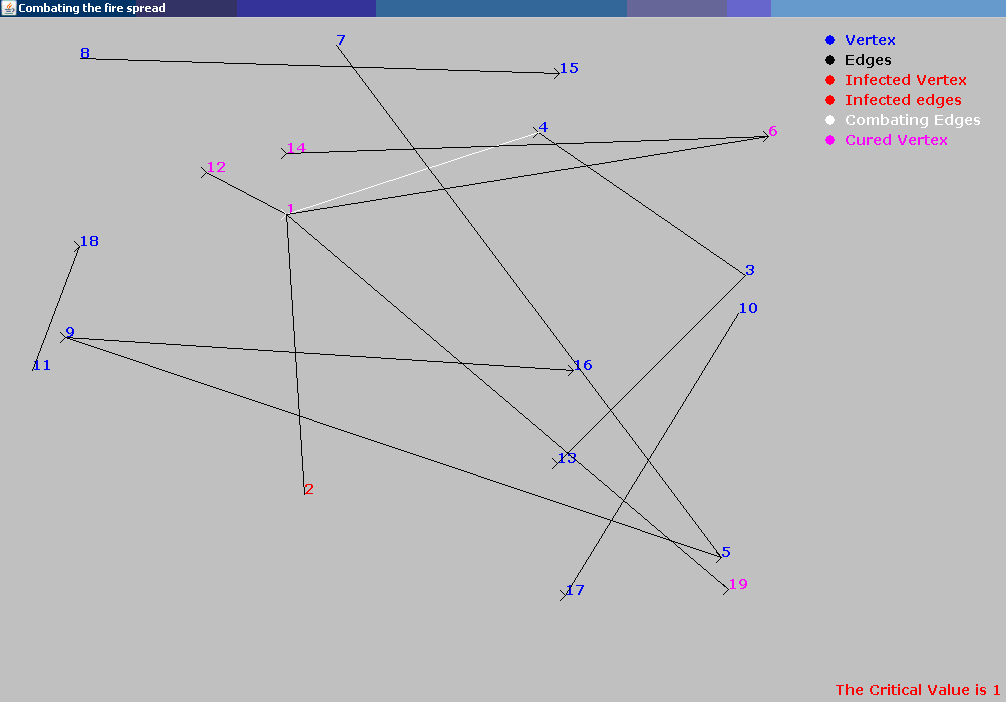


Fig. S9: β-alanine metabolism; Combat analysis for infection start site = L-aspartate


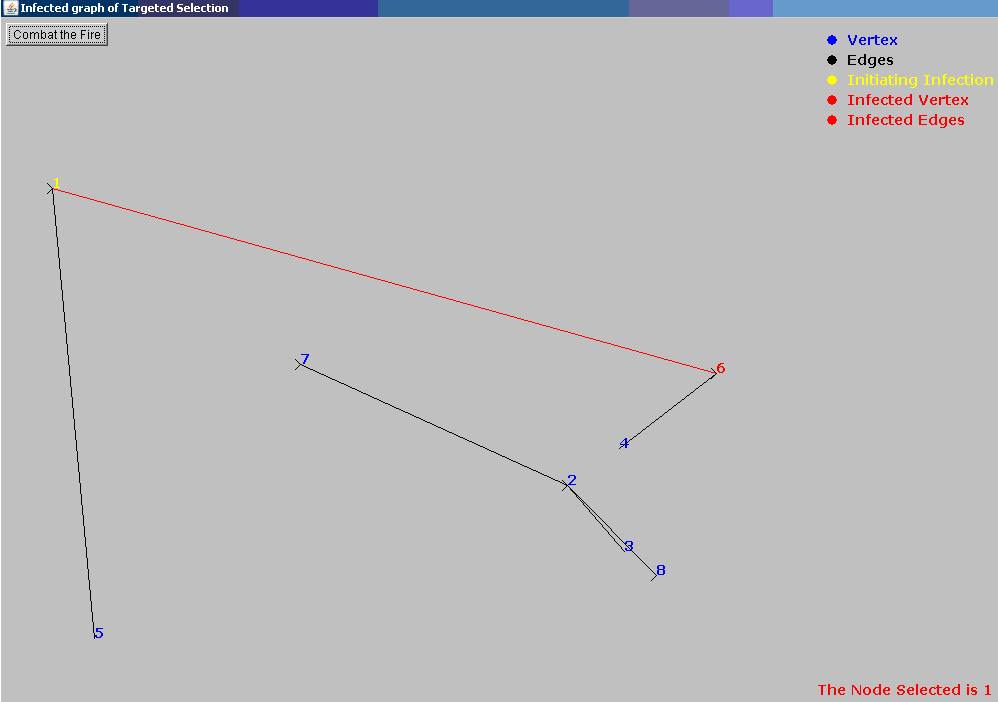


Fig. S10: Taurine and hypotaurine metabolism; Infection start site = 3-sulfino-L-alanine


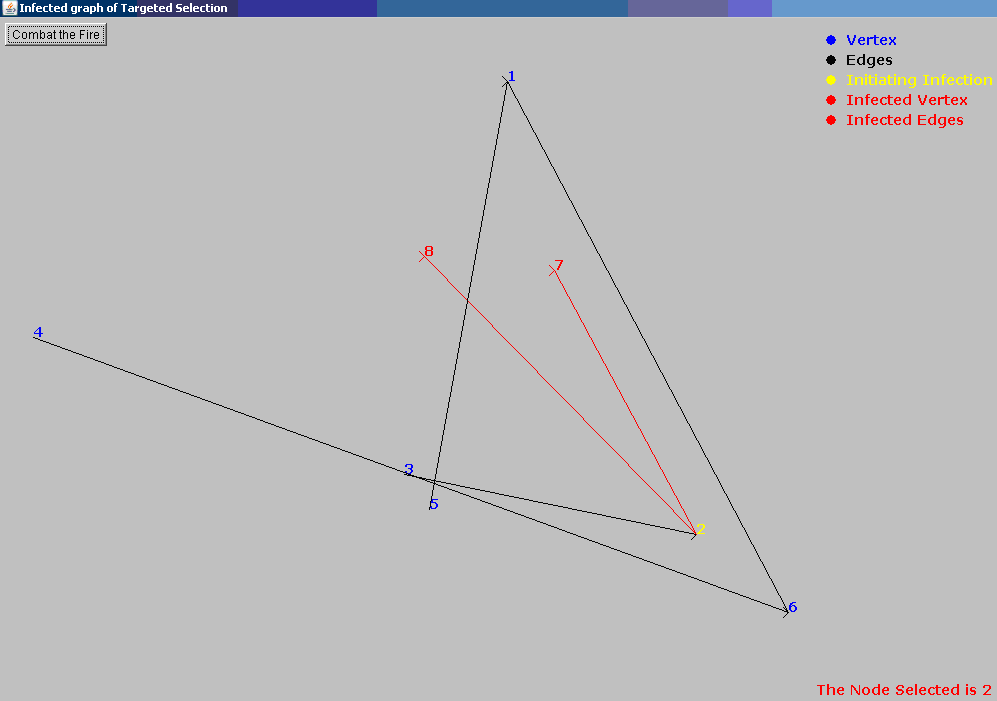


Fig. S11: Taurine and hypotaurine metabolism; Infection start site = taurine


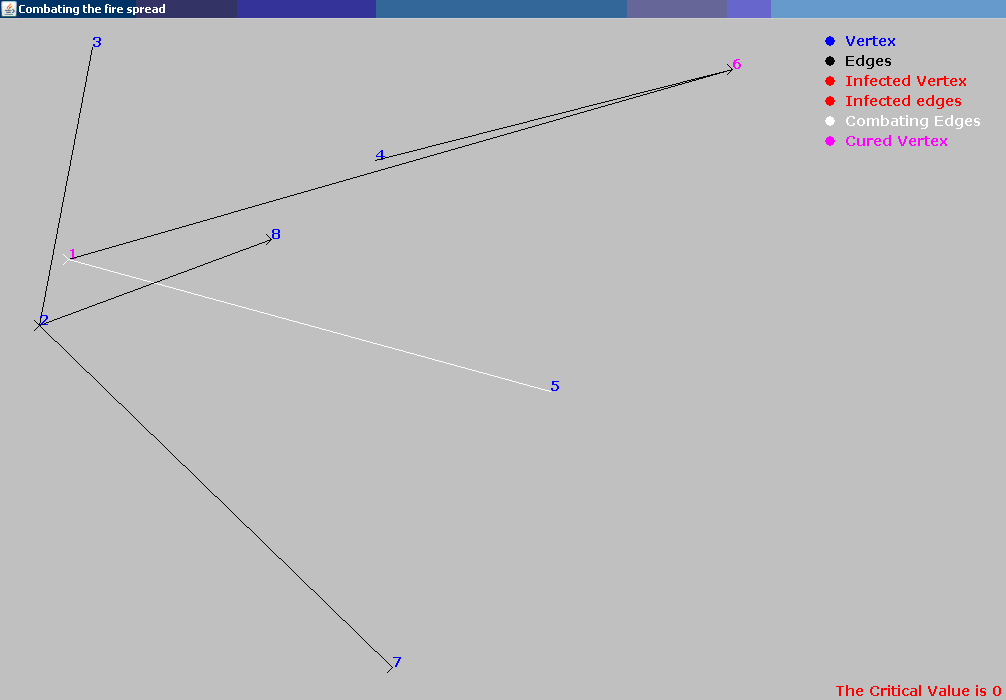


Fig. S12: Taurine and hypotaurine metabolism; Combat analysis for infection start site = 3-sulfino-L-alanine


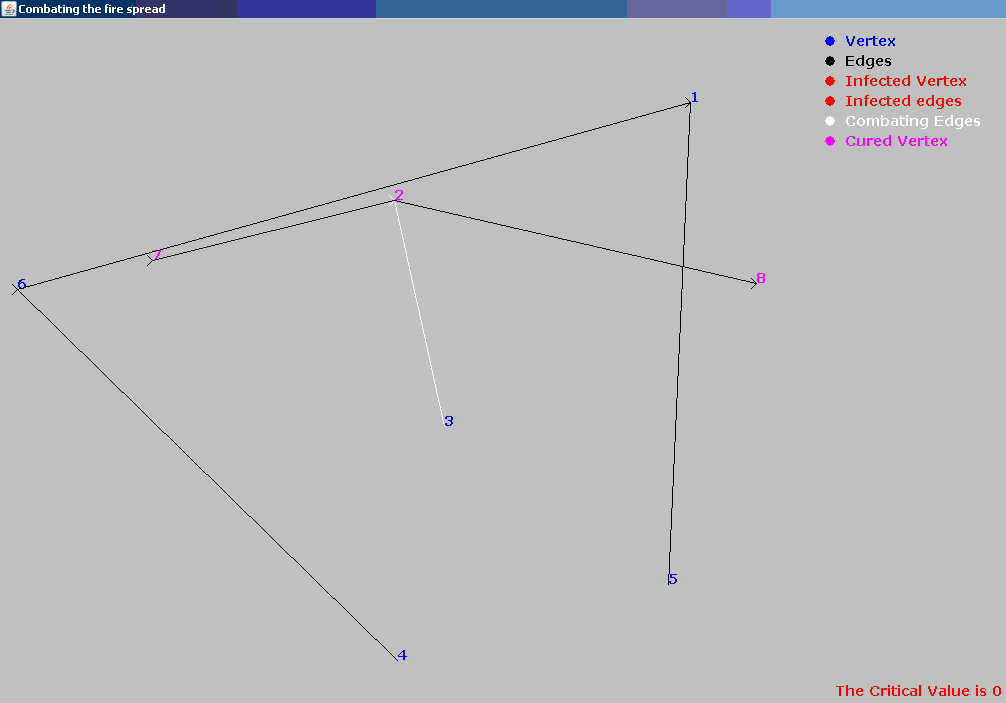


Fig. S13: Taurine and hypotaurine metabolism; Combat analysis for infection start site = taurine


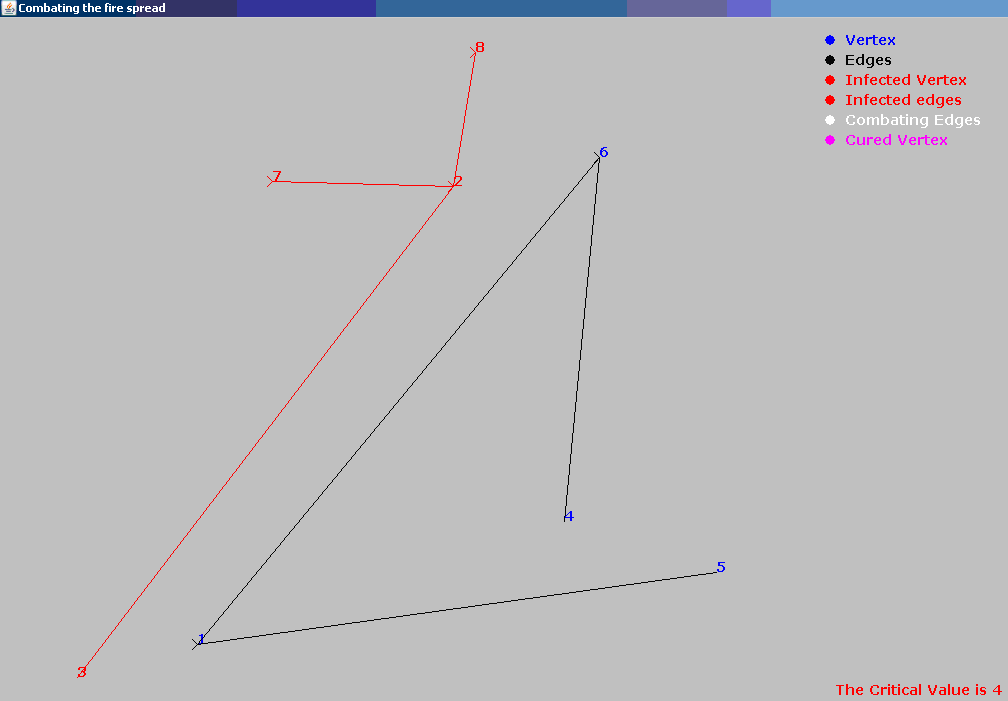


Fig. S14: Taurine and hypotaurine metabolism; Combat analysis for infection start site = L-cysteate


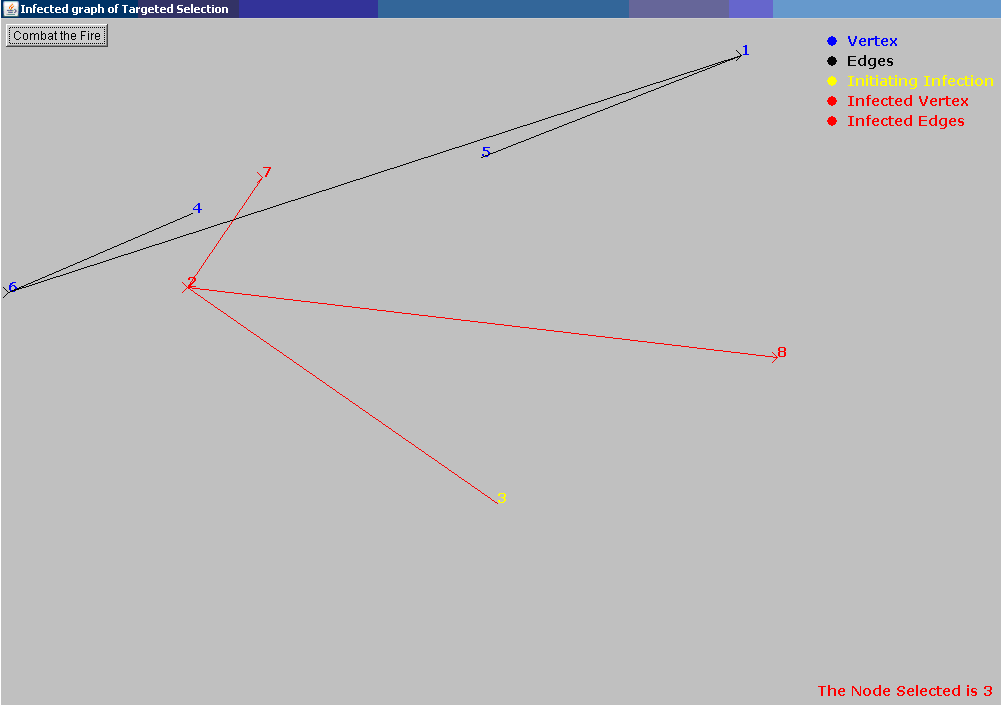


Fig. S15: Taurine and hypotaurine metabolism; Infection start site = L-cysteate


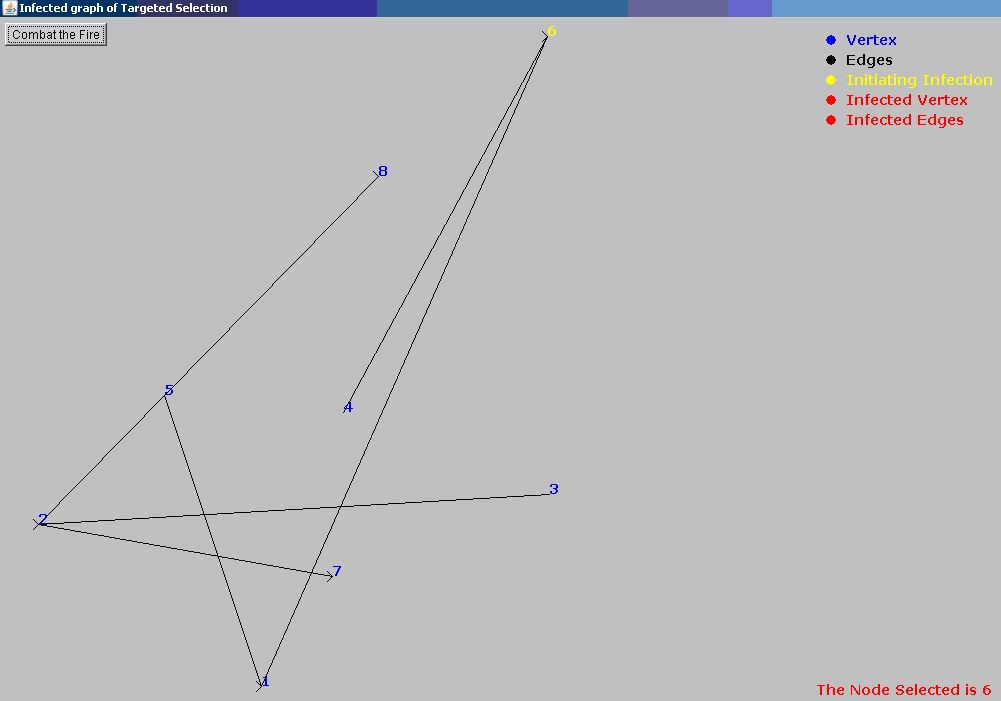


Fig. S16: Taurine and hypotaurine metabolism; Infection start site = hypotaurine

**Modeling infection spread in butanoate metabolism**

In continuation with our work in main manuscript, we had restricted ourselves in explaining infection spread in only 3 metabolic pathways. We discuss the infection spread in 4^th^ metabolic pathway here. In butanoate metabolism, only two runs for infection spread simulation are possible, as 4 - aminobutanoate and L-glutamate could act as start site for infection [Al-Mutairi et al., 2007]. Initiating with 4 – aminobutanoate (d^+^ = 1; BP = 1) as infection start site, succinate – semialdehyde (d^+^ = 1; BP = 1) gets infected affecting succinate (d^+^ = 0) that terminates the growth of infection any further (Section B Fig. 25 in Supplementary Information). Thus, M = 19;N = 0; P = 3; I_act_ = 4; I_pas_ = 3;Np = 0;C_act_ = 0:25;C_pas_ = 0.33; I = 19; q = 0; β = 0; α = 1; µ = 0.15; ɛ = 0; I^l^ = 19. Thus, dP/dt = 8.54; dI_act_/dt = 226.56; I_pas_/dt = -1.6; ρ_4_ = 0. Lastly, in case of butanoate metabolism, 4 – aminobutanoate (d^-^ = 1; CP = 1) has a combat edge with L – glutamate (d^-^ = 0) having no ability to perform any combat action. For the 2^nd^ run, L-glutamate (d^+^ = 1; BP = 1) is selected as start site for infection resulting in infecting succinate - semialdehyde, which in turn spreads the infection further (Section B Fig. 26 in Supplementary Information). Thus, M = 19;N = 0; P = 1; I_act_ = 1; I_pas_ = 3;Np = 0;C_act_ = 1;C_pas_ = 1; I = 19; q = 0; β = 0; α = 1; µ = 0.05; ɛ = 0; I^l^ = 15. Thus, dP/dt = 18.95; dI_act_/dt = 17.95; I_pas_/dt = -1.05; ρ_4_ = 4 (Section B Figs. 27, 28 in Supplementary Information) [Jain, 2012].


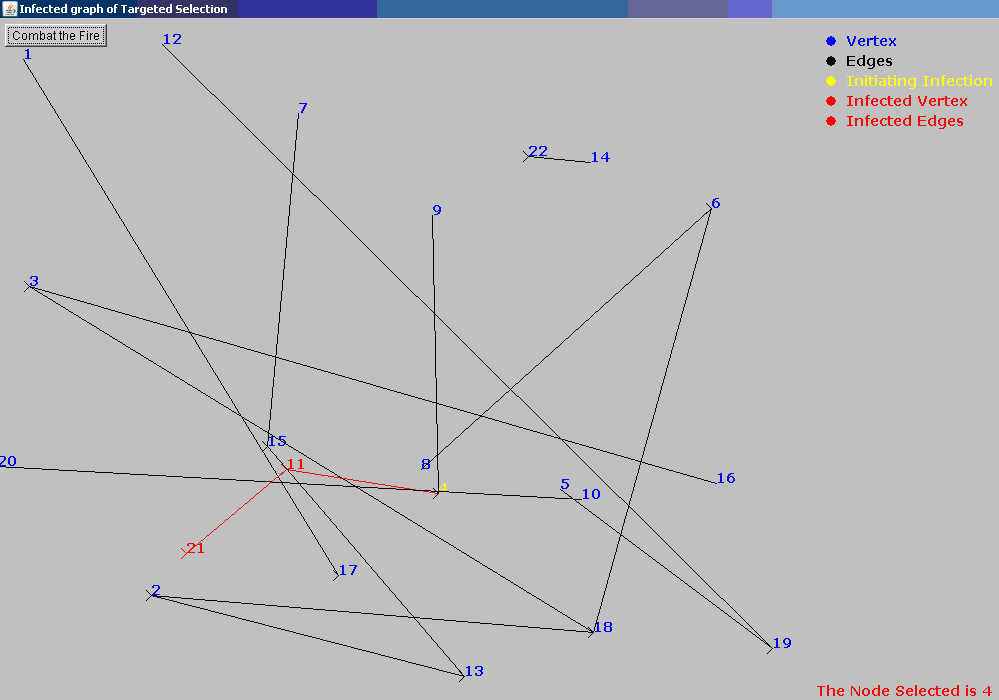


Fig. S17: Butanoate metabolism; Infection start site = 4-aminobutanoate


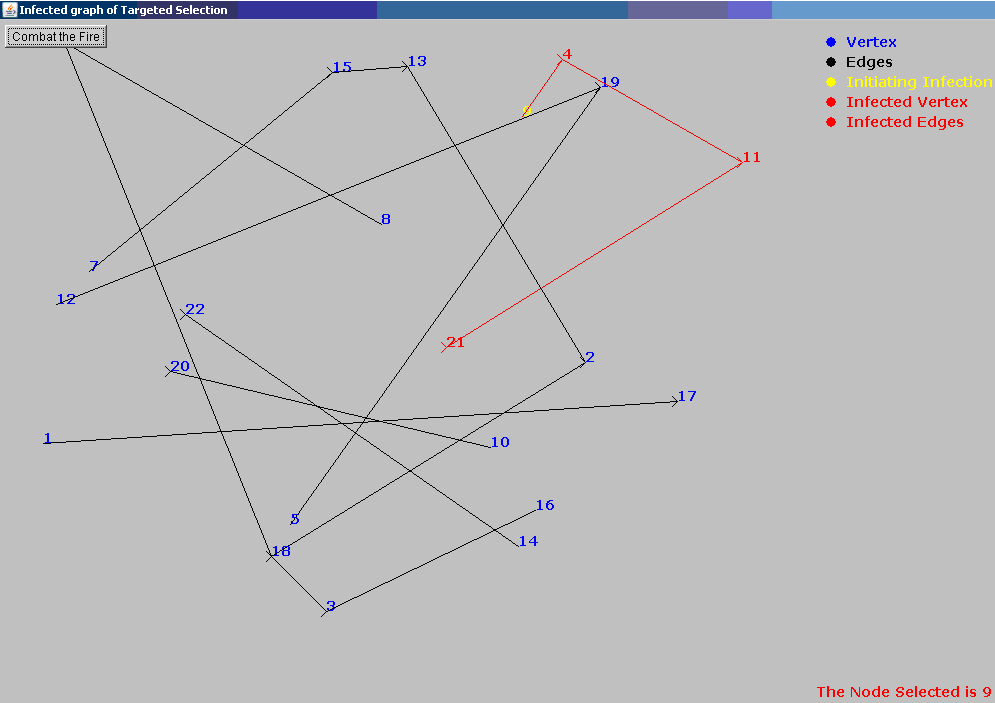


Fig. S18: Butanoate metabolism; Infection start site = L-glutamate


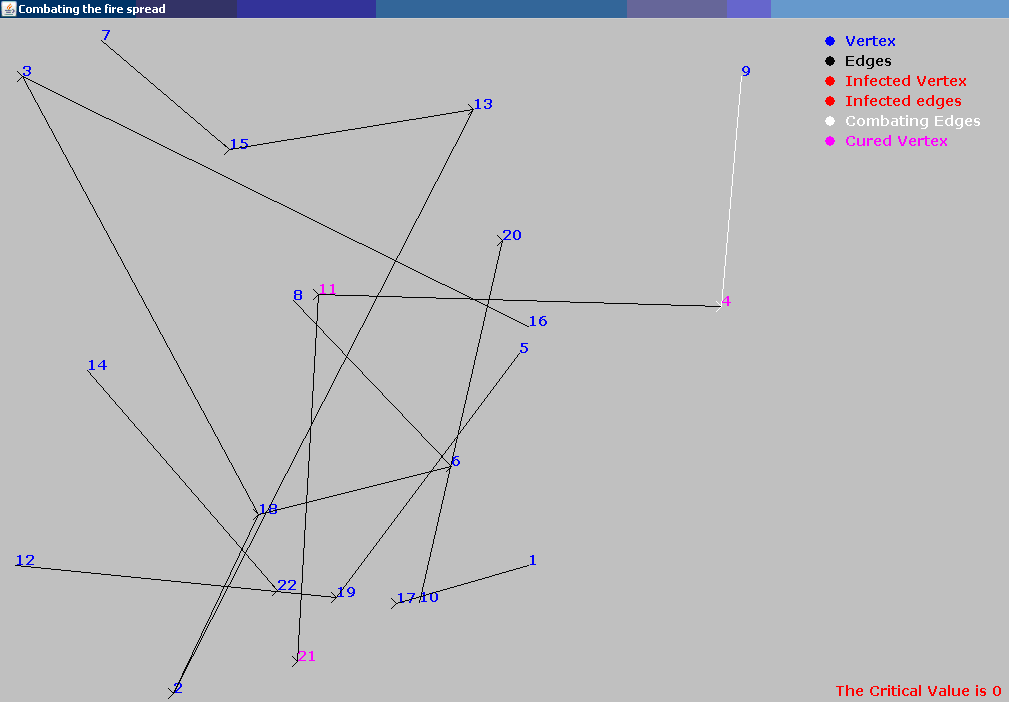


Fig. S19: Butanoate metabolism; Combat analysis for infection start site = 4-aminobutanoate


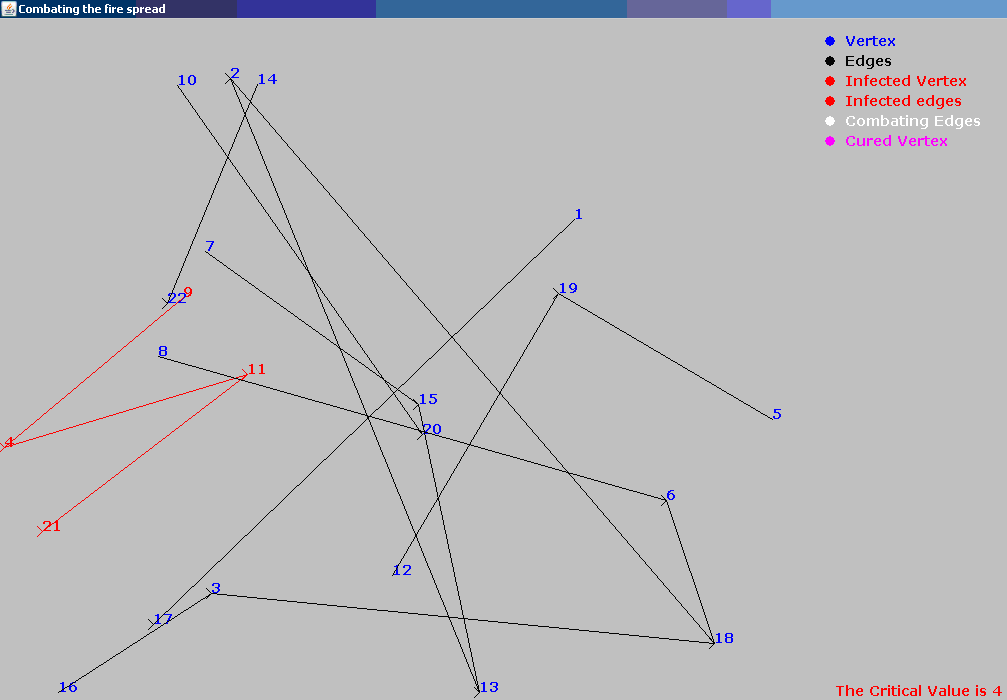


Fig. S20: Butanoate metabolism; Combat analysis for infection start site = L-glutamate

**Section S3**

(I)

Feedback analysis


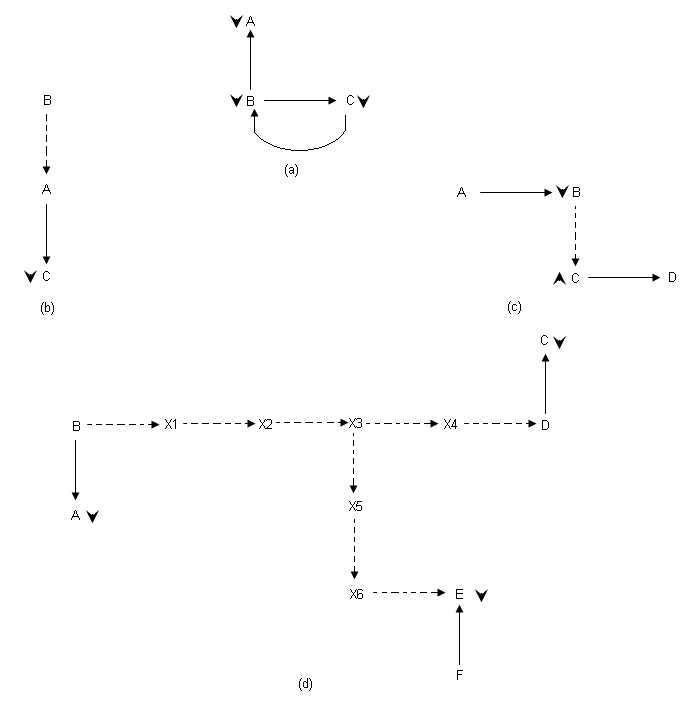


Figure S21 : (a) Glutamate metabolism: 2-oxoglutarate [A], L-glutamate [B], L-glutamine [C] (b) Beta-alanine metabolism: acetyl CoA [A], malonate semialdehyde [B], malonyl CoA [C] (c) Taurine and hypotaurine metabolism: 3-sulfino-L-alanine [A], hypotaurine [B], taurine [C], 5-glutaryl-taurine [D] (d) Butanoate metabolism: L-glutamate [A], 4-aminobutanoate [B], 4-hydroxybutanoate [X1], 3-butanoyl CoA [X2], crotonyl CoA [X3], glutaconyl CoA [X4], 2-hydroxyglutarate [D], 2-oxoglutarate [C], (S)-3-hydroxybutanoyl CoA [X5], acetoacetyl CoA [X6], acetyl CoA [E], (S)-3-hydroxy-3-methyl glutaryl CoA [F]

**Detecting feedback reactions in butanoate metabolism**

In continuation with our work in main manuscript, we had restricted ourselves in explaining the detection methodology for feedback reactions in only 3 metabolic pathways. We discuss the same in the 4^th^ metabolic pathway here. Lastly, for butanoate metabolism, three reactions were found to be having properties of feedback links, namely, 4 - aminobutanoate + 2 - oxoglutarate succinatesemialdehyde + α - glutarate; 2 - hydroxyglutarate + FAD 2 - oxoglutarate + FADH2 and (S) - 3 - hydroxy - 3 - methylglutarylCoA acetylCoA + acetoacetate, for which sequential links are found (Section C Fig. 29d in Supplementary Information). For d^+^(n_j+1_) = 3(4 - aminobutanoate); d^+^(n_j+1_) = 2(2 - hydroxyglutarate); d^+^(n_j+1_) = 3((S) - 3 - hydroxy - 3 - methylglutarylCoA); k = 1, links are found between 4-aminobutanoate;L-glutamate, 2-hydroxyglutarate; 2-oxoglutarate and (S) - 3 - hydroxy - 3 - methylglutarylCoA; acetylCoA respectively. For validation, we found dx_1_/dt (4 - aminobutanoate) = 1; dx_2_/dt (L - glutamate) = -1:18E-19; dx_1_/dt (2 - oxoglutarate) = 1; dx_2_/dt (2 -oxoglutarate) = -1.12E-20; dx_1_/dt ((S)-3-hydroxy-3-methylglutarylCoA) = 1; dx_2_/dt (acetylCoA) = -1.23E-20. Negative gradients in terms of concentration was found for L-glutamate; 2-oxoglutarate and 2-oxoglutarate; acetylCoA, showing possibility of feedback links [Burton et al., 1992].

**Section S4**

Local sensitivity analysis

Glutamate metabolism

Table S1: Target function, non-constant concentration of species, variable, initial concentration


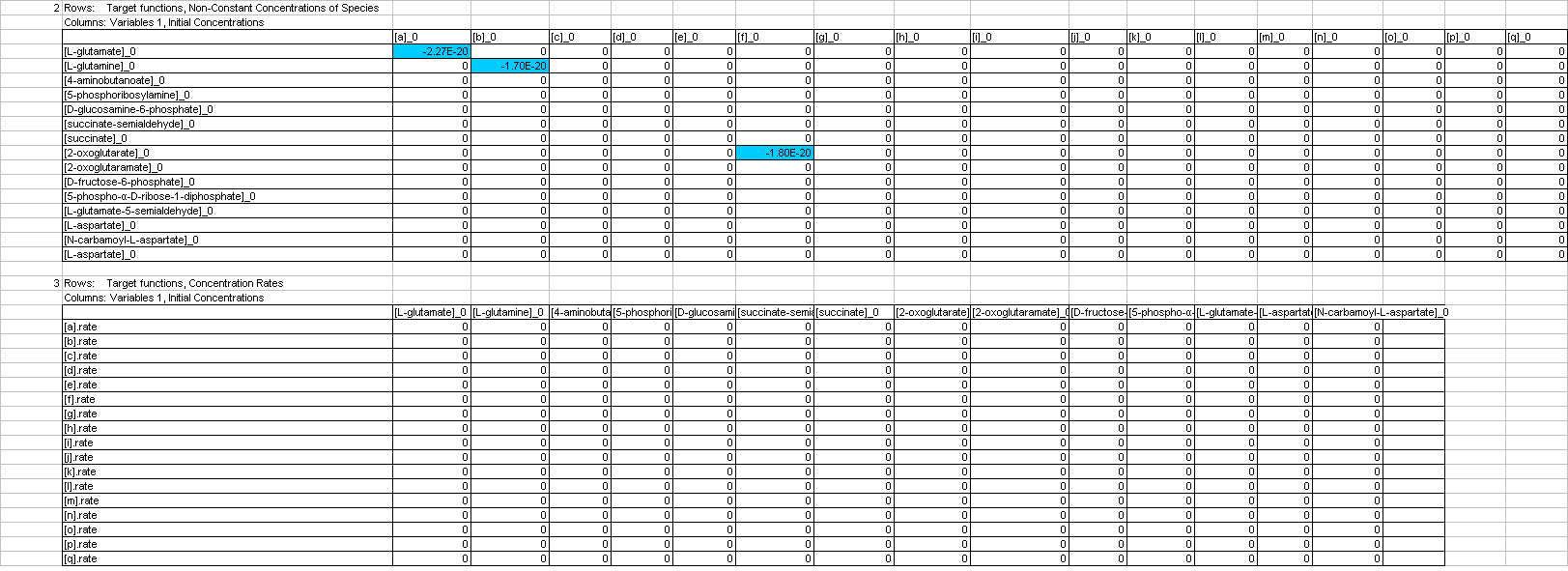


Beta-alanine metabolism

Table S2: Target function, concentration fluxes of reactions, variable, initial concentration


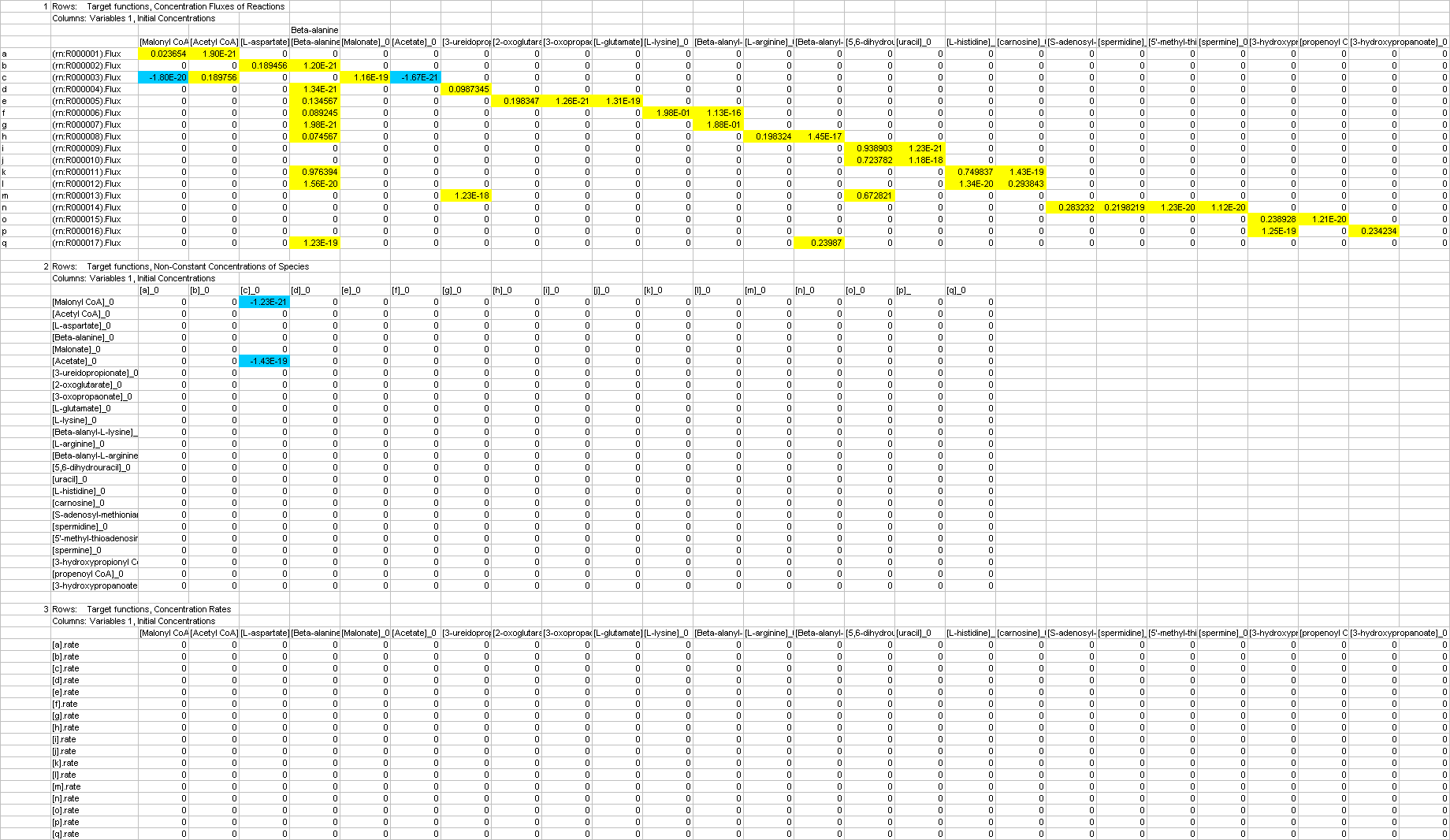


Taurine and hypotaurine metabolism

Table S3: Target function, concentration fluxes of reactions, variable, initial concentration


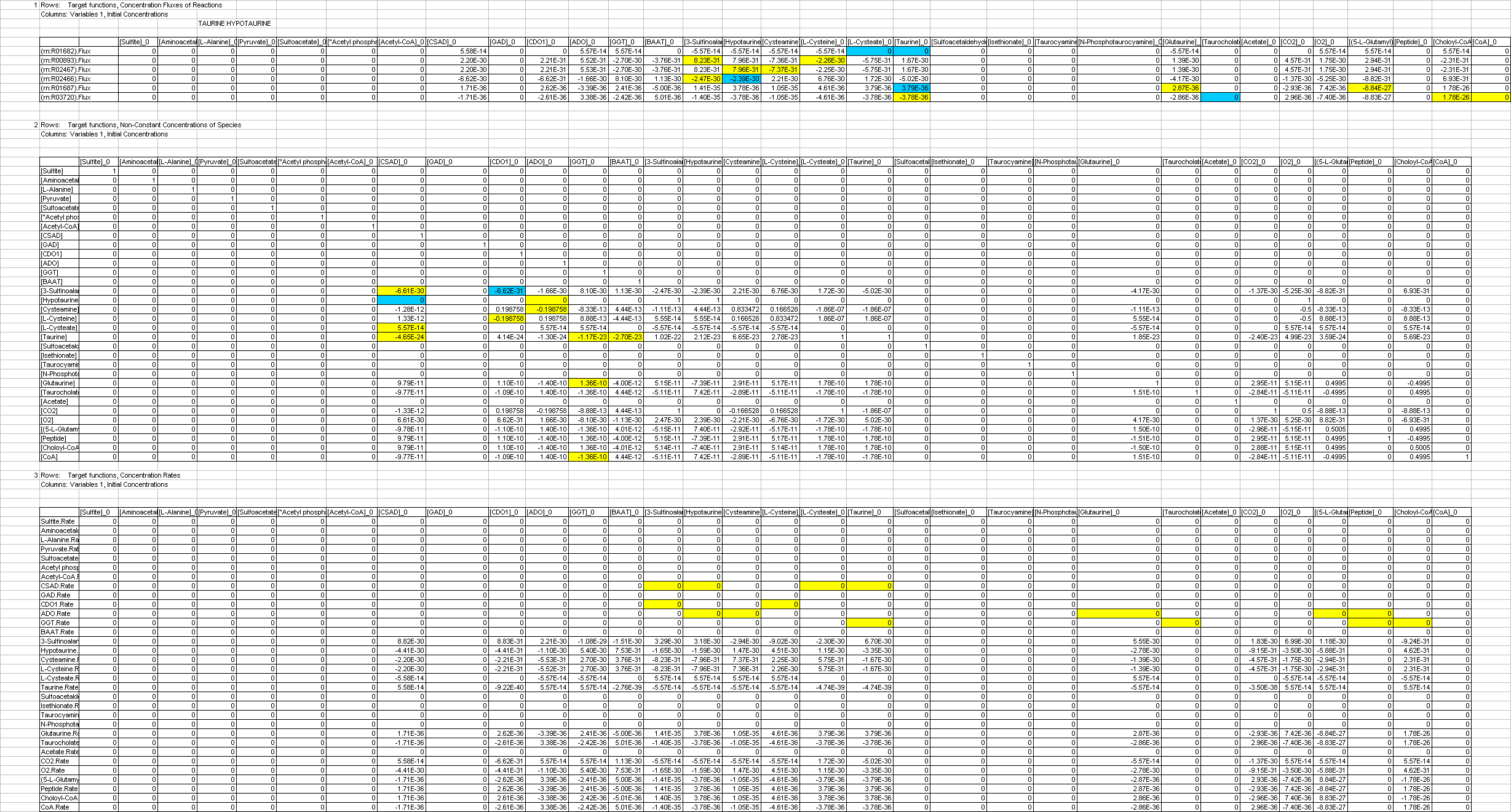


**Performing local sensitivity analysis in Butanoate metabolism**

In continuation with our work in main manuscript, we had restricted ourselves in explaining the local sensitivity analysis in only 3 metabolic pathways. We discuss the same in the 4^th^ metabolic pathway here. Lastly, in butanoate metabolism, (Section D(IV) Table 6 in Supplementary Information), with acetylCoA ~ -1.23E-20µM in reaction flux ‘(rn:R00009).Flux’, L-glutamate ~ -1.18E-19µM in reaction flux ‘(rn:R00011).Flux’ and 2 - oxoglutarate ~ -1.12E -20µM in reaction flux ‘(rn:R00015).Flux’ have decreasing negative gradients, whereas in (Section D(IV) Table 6 in Supplementary Information), these metabolites again display negative gradient of concentration of -1.19E-20µM;-1.23E -19µM;-1.12E -19 for genes i; k; o respectively. Furthermore, validating this result with fire spread model, 4 - aminobutanoate ~ 1.23E-18 in ‘(rn:R00003).Flux’ and ~ 0.9843984 in ‘(rn:R00011).Flux’ respectively display a positive decreasing gradient, which is obvious, due to its presence in a reaction [Rabitz, 1983]. In this section we looked into the metabolic pathways from a local structure point of view, which suffers from several disadvantages such as, their role to investigate the model behavior in the immediate region around the nominal parameter values and only consider changes to one parameter at a time, while all other parameters are fixed to their nominal values. Thus, to understand this situation, we perform global sensitivity analysis of infected metabolic pathways [Gylvin et al., 2004].

Table S4: Target function, concentration fluxes of reactions, variable, initial concentration


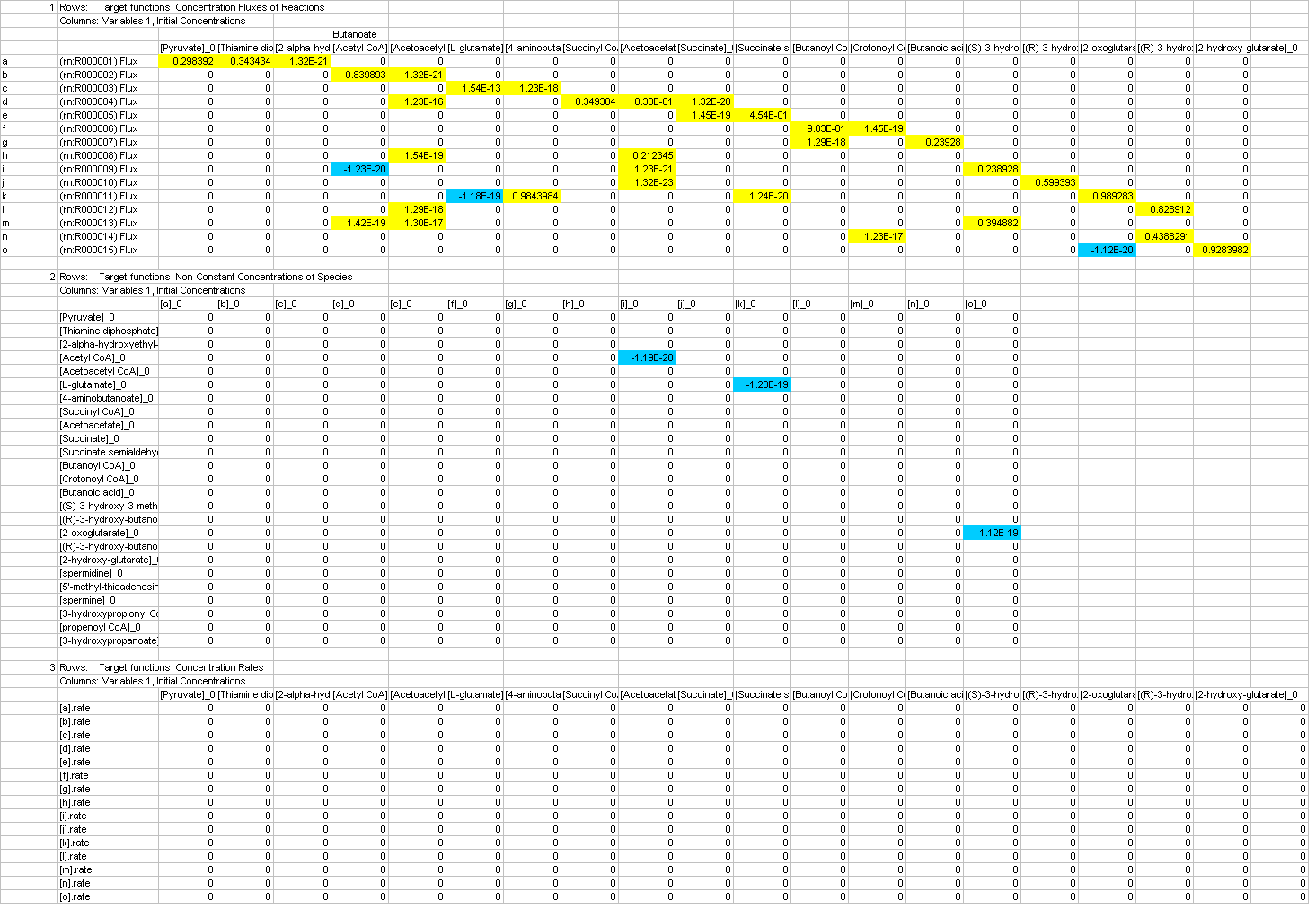


**Section S5**

(SI)

Global sensitivity analysis

Beta-alanine metabolism


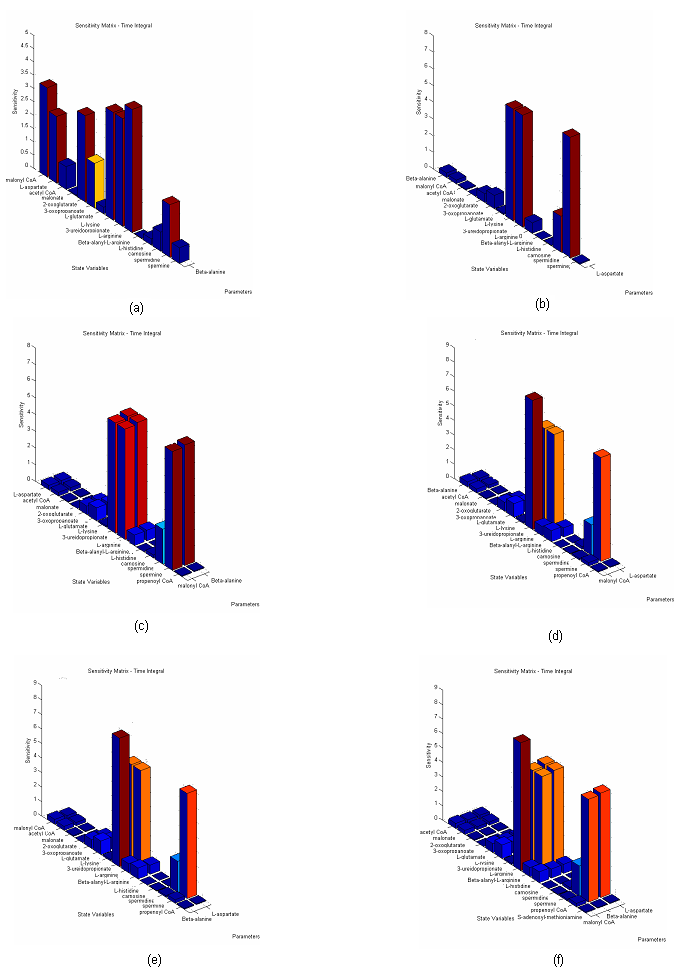


Fig. S22: Global sensitivity analysis of beta-alanine metabolism for (a) beta-alanine (b) L-aspartate (c) malonyl CoA, beta-alanine (d) malonyl CoA, L-aspartate (e) beta-alanine, L-aspartate (f) beta-alanine, L-aspartate, malonyl CoA

(SII)

Taurine and hypotaurine metabolism


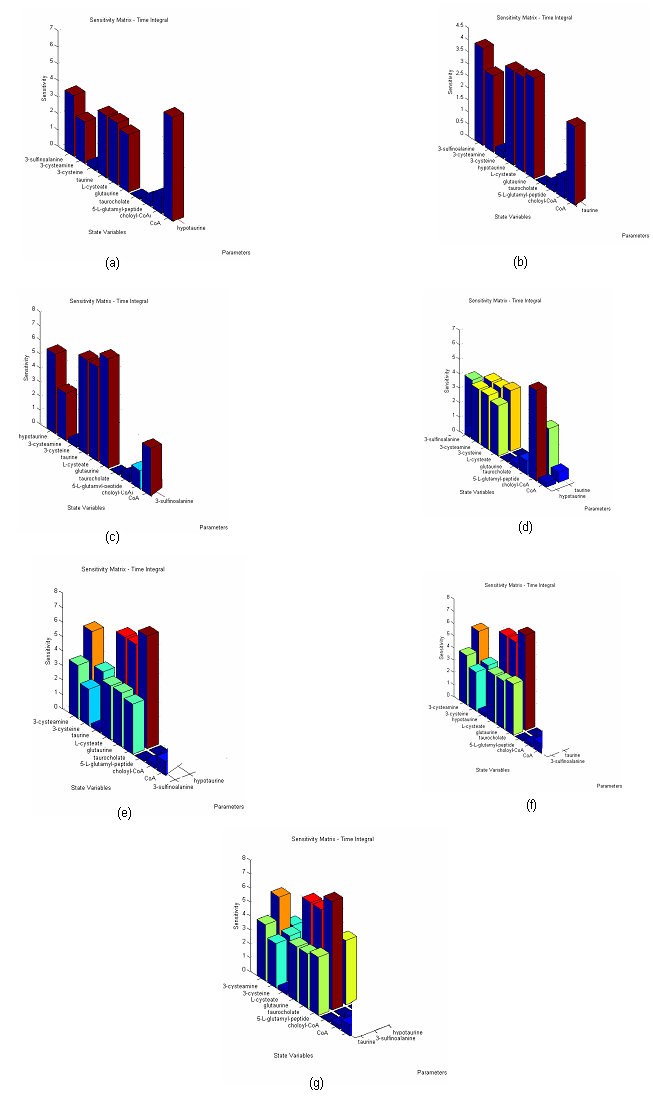


Fig. S23: Global sensitivity analysis of taurine-hypotaurine metabolism for (a) hypotaurine (b) taurine (c) 3-sulfino-L-alanine (d) hypotaurine, taurine (e) 3-sulfino-L-alanine, hypotaurine (f) 3-sulfino-L-alanine, taurine (g) 3-sulfino-L-alanine, taurine, hypotaurine

(SIII)

**Performing global sensitivity analysis in Butanoate metabolism**

In continuation with our work in main manuscript, we had restricted ourselves in explaining the global sensitivity analysis in only 3 metabolic pathways. We discuss the same in the 4^th^ metabolic pathway here. Lastly, in butanoate metabolism, considering acetylCoA, maximal effect is seen on succinylCoA, succinate, whereas for L-glutamate effect is seen on acetylCoA and 2α-hydroxyethyl-dipphosphate, for 2-oxoglutarate maximal effect is seen on acetylCoA, succinylCoA, succinate, succinate-semialdehyde. Considering acetylCoA and L-glutamate, group effect is identified in succinylCoA and succinate, for acetylCoA and 2-oxoglutarate, effect is observed in 4 - aminobutanoate, succinylCoA and succinate, whereas for L - glutamate and 2 - oxoglutarate, effect is observed in succinate - semialdehyde, butanoylCoA and crotonylCoA. Finally, considering all the three metabolites, effect is observed in succinate - semialdehyde, butanoylCoA and crotonylCoA respectively. Thus, the fire spread model suggested an effect of over 15 metabolites due to infection spread, which is similar to our results shown using global sensitivity analysis validating our results that we found previously (Section E(III) Figs. 32 a-g in Supplementary Information) [Sobol, 1993].


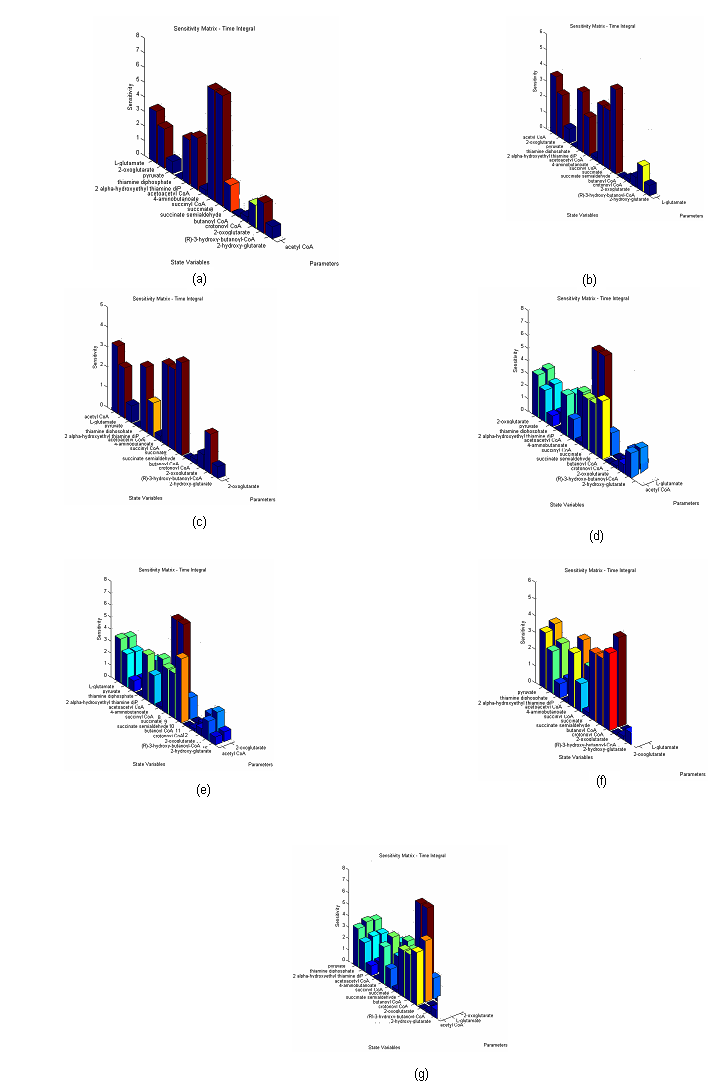


Fig. S24: Global sensitivity analysis of butanoate metabolism for (a) acetyl CoA(b) L-glutamate (c) 2-oxoglutarate (d) acetyl CoA, L-glutamate (e) acetyl CoA, 2-oxoglutarate (f) L-glutamate, 2-oxoglutarate (g) acetyl CoA, 2-oxoglutarate, L-glutamate

**Section S6**

(SI)

Critical value analysis

Table S5: Identification of infected, cured and un-cured metabolites

| **Metabolic Pathway** | **Infection start site** | **Metabolites infected** | **Metabolites cured** | **Metabolites not cured** |
| --- | --- | --- | --- | --- |
| Glutamate metabolism | L-glutamate | L-glutamate,  2-oxoglutarate,  Succinate,  L-glutaryl-tRNA Glu,  4-aminobutanoate,  Glutathione disulfide,  Succinate semialdehyde,  NAD^+^,  Glutathione,  L-glutamine,  Carbamoyl phosphate,  N-acetyl-D-glucosamine-6-phosphate,  D-glucosamine-6-phosphate,  Glutamyl tRNA,  γ-L-glutamyl-L-cysteine,  GMP | L-glutamate,  2-oxoglutarate,  Succinate,  L-glutaryl-tRNA Glu,  4-aminobutanoate,  Glutathione disulfide,  Succinate semialdehyde,  NAD^+^,  Glutathione,  L-glutamine,  Carbamoyl phosphate,  N-acetyl-D-glucosamine-6-phosphate,  D-glucosamine-6-phosphate,  Glutamyl tRNA,  γ-L-glutamyl-L-cysteine,  GMP | NIL |
|  | 4-aminobutanoate | 4-aminobutanoate,  Succinate semialdehyde,  Succinate | 4-aminobutanoate,  Succinate semialdehyde,  Succinate | NIL |
| β-alanine metabolism | β-alanine | β-alanine,  3-oxopropanoate,  β-aminopropion aldehyde,  β-alanyl-N-pi-methyl-L-histidine,  1,3-diaminopropane | β-alanine,  3-oxopropanoate,  β-aminopropion aldehyde,  β-alanyl-N-pi-methyl-L-histidine,  1,3-diaminopropane | NIL |
|  | L-aspartate | L-aspartate,  β-alanine,  3-oxopropanoate,  β-aminopropion aldehyde,  β-alanyl-N-pi-methyl-L-histidine,  1,3-diaminopropane | β-alanine,  3-oxopropanoate,  β-aminopropion aldehyde,  β-alanyl-N-pi-methyl-L-histidine,  1,3-diaminopropane | L-aspartate |
| Taurine and Hypotaurine metabolism | 3-sulfino-L-alanine | 3-sulfino-L-alanine,  Hypotaurine | 3-sulfino-L-alanine,  Hypotaurine | NIL |
|  | Taurine | Taurine,  Taurocholate,  5-glutamyl-taurine | Taurine,  Taurocholate,  5-glutamyl-taurine | NIL |
|  | L-cysteate | L-cysteate,  Taurine,  Taurocholate,  5-glutamyl-taurine | NIL | L-cysteate,  Taurine,  Taurocholate,  5-glutamyl-taurine |
|  | Hypotaurine | NIL | NIL | NIL |
| Butanoate metabolism | 4-aminobutanoate | 4-aminobutanoate,  Succinate semialdehyde,  Succinate | 4-aminobutanoate,  Succinate semialdehyde,  Succinate | NIL |
|  | L-glutamate | L-glutamate,  4-aminobutanoate,  Succinate semialdehyde,  Succinate | NIL | L-glutamate,  4-aminobutanoate,  Succinate semialdehyde,  Succinate |

(SII)

Table S6: Critical value analysis

| **SL. No.** | **Metabolic Pathway** | **Metabolic Pathway** | **Minimum Critical Value** | **Maximum Critical Value** | **Avg. Critical Value** |
| --- | --- | --- | --- | --- | --- |
| 1 | Carbohydrate metabolism | hsa00010 | 1 | 4 | 4.59375 |
| 2 |  | hsa00020 | 1 | 4 |  |
| 3 |  | hsa00030 | 1 | 4 |  |
| 4 |  | hsa00040 | 1 | 5 |  |
| 5 |  | hsa00052 | 1 | 2 |  |
| 6 |  | hsa00053 | 2 | 2 |  |
| 7 |  | hsa00056 | 1 | 4 |  |
| 8 |  | hsa00500 | 1 | 3 |  |
| 9 |  | hsa00562 | 1 | 2 |  |
| 10 |  | hsa00620 | 1 | 2 |  |
| 11 |  | hsa00630 | 1 | 4 |  |
| 12 |  | hsa00640 | 1 | 7 |  |
| 13 |  | hsa00650 | 1 | 4 |  |
| 14 | Amino acid metabolism | hsa00260 | 1 | 5 | 6.227273 |
| 15 |  | hsa00280 | 1 | 4 |  |
| 16 |  | hsa00790 | 1 | 3 |  |
| 17 |  | hsa00300 | 3 | 5 |  |
| 18 |  | hsa00310 | 1 | 9 |  |
| 19 |  | hsa00330 | 1 | 9 |  |
| 20 |  | hsa00340 | 1 | 10 |  |
| 21 |  | hsa00350 | 1 | 25 |  |
| 22 |  | hsa00360 | 1 | 8 |  |
| 23 |  | hsa00380 | 1 | 35 |  |
| 24 |  | hsa00400 | 6 | 6 |  |
| 25 | Lipid metabolism | hsa00061 | 1 | 22 | 6.892857 |
| 26 |  | hsa00062 | 25 | 25 |  |
| 27 |  | hsa00071 | 1 | 4 |  |
| 28 |  | hsa00072 | 2 | 2 |  |
| 29 |  | hsa00100 | 1 | 14 |  |
| 30 |  | hsa00120 | 1 | 8 |  |
| 31 |  | hsa00140 | 1 | 22 |  |
| 32 |  | hsa00561 | 1 | 2 |  |
| 33 |  | hsa00564 | 1 | 4 |  |
| 34 |  | hsa00565 | 1 | 4 |  |
| 35 |  | hsa00590 | 0 | 35 |  |
| 36 |  | hsa00591 | 2 | 6 |  |
| 37 |  | hsa00592 | 2 | 3 |  |
| 38 |  | hsa00600 | 1 | 2 |  |
| 39 | Energy metabolism | hsa00680 | 2 | 3 | 2.2 |
| 40 |  | hsa00710 | 1 | 4 |  |
| 41 |  | hsa00720 | 1 | 4 |  |
| 42 |  | hsa00910 | 1 | 2 |  |
| 43 |  | hsa00920 | 2 | 2 |  |
| 44 | Metabolism of Cofactors and Vitamins | hsa00730 | 1 | 2 | 2.8 |
| 45 |  | hsa00740 | 2 | 2 |  |
| 46 |  | hsa00750 | 2 | 2 |  |
| 47 |  | hsa00790 | 1 | 7 |  |
| 48 |  | hsa00860 | 1 | 8 |  |

**Section S7**

Validation using step changes in input dataset

Table S7: Perturbations in β-alanine metabolism

| Metabolites | Initial Conc. | Perturbation 1 | Perturbation 2 | Perturbation 3 | Perturbation 4 |
| --- | --- | --- | --- | --- | --- |
| Malonyl-CoA | 0.4 | 0.3 | 0.2 | 0.5 | 0.6 |
| Acetyl-CoA | 0.3 | 0.2 | 0.1 | 0.4 | 0.5 |
| L-aspartate | 0.2 | 0.1 | 0.05 | 0.3 | 0.4 |
| β-alanine | 1.0 | 0.8 | 0.7 | 1.2 | 0.4 |
| Malonate | 0.2 | 0.1 | 0.05 | 0.3 | 0.4 |
| Acetate | 0.2 | 0.1 | 0.05 | 0.3 | 0.4 |
| 3-ureidopropionate | 0.4 | 0.3 | 0.2 | 0.5 | 0.6 |
| 2-oxoglutarate | 0.2 | 0.1 | 0.05 | 0.3 | 0.4 |
| 3-oxopropionate | 0.2 | 0.1 | 0.05 | 0.3 | 0.4 |
| L-glutamate | 0.2 | 0.1 | 0.05 | 0.3 | 0.4 |
| L-lysine | 0.4 | 0.3 | 0.2 | 0.5 | 0.6 |
| β-alanyl-L-lysine | 0.4 | 0.3 | 0.2 | 0.5 | 0.6 |
| L-arginine | 0.2 | 0.1 | 0.05 | 0.3 | 0.4 |
| 5,6-dihydrouracil | 0.6 | 0.5 | 0.4 | 0.7 | 0.8 |
| Uracil | 0.4 | 0.3 | 0.2 | 0.5 | 0.6 |
| L-histidine | 0.4 | 0.3 | 0.2 | 0.5 | 0.6 |
| Carnosine | 0.4 | 0.3 | 0.2 | 0.5 | 0.6 |
| S-adenosyl methioninamine | 0.2 | 0.1 | 0.05 | 0.3 | 0.4 |
| Spermidine | 0.2 | 0.1 | 0.05 | 0.3 | 0.4 |
| 5’-methyl thioadenosine | 0.2 | 0.1 | 0.05 | 0.3 | 0.4 |
| Spermine | 0.2 | 0.1 | 0.05 | 0.3 | 0.4 |
| 3-hydroxypropionyl-CoA | 0.4 | 0.3 | 0.2 | 0.5 | 0.6 |
| Propenoyl-CoA | 0.2 | 0.1 | 0.05 | 0.3 | 0.4 |
| β-alanyl-L-arginine | 0.4 | 0.3 | 0.2 | 0.5 | 0.6 |

Table S8: Perturbations in taurine and hypotaurine metabolism

| Metabolites | Initial Conc. | Perturbation 1 | Perturbation 2 | Perturbation 3 | Perturbation 4 |
| --- | --- | --- | --- | --- | --- |
| L-cysteate | 0.4 | 0.3 | 0.2 | 0.5 | 0.6 |
| taurine | 0.4 | 0.3 | 0.2 | 0.5 | 0.6 |
| L-cysteine | 0.2 | 0.1 | 0.05 | 0.3 | 0.4 |
| 3-sulfinoalanine | 0.6 | 0.5 | 0.4 | 0.7 | 0.8 |
| Cysteamine | 0.4 | 0.3 | 0.2 | 0.5 | 0.6 |
| hypotaurine | 0.4 | 0.3 | 0.2 | 0.5 | 0.6 |
| (5-L-glutamyl)-peptide | 0.4 | 0.3 | 0.2 | 0.5 | 0.6 |
| glutaurine | 0.2 | 0.1 | 0.05 | 0.3 | 0.4 |
| choloyl-CoA | 0.2 | 0.1 | 0.05 | 0.3 | 0.4 |
| taurocholate | 0.2 | 0.1 | 0.05 | 0.3 | 0.4 |
| CoA | 0.4 | 0.3 | 0.2 | 0.5 | 0.6 |

Table S9: Perturbations in butanoate metabolism

| Metabolites | Initial Conc. | Perturbation 1 | Perturbation 2 | Perturbation 3 | Perturbation 4 |
| --- | --- | --- | --- | --- | --- |
| Pyruvate | 0.2 | 0.1 | 0.05 | 0.3 | 0.4 |
| Thiamin diphosphate | 0.2 | 0.1 | 0.05 | 0.3 | 0.4 |
| 2-(alpha-Hydroxyethyl)thiamine diphosphate | 0.6 | 0.5 | 0.4 | 0.7 | 0.8 |
| CoA | 0.4 | 0.3 | 0.2 | 0.5 | 0.6 |
| Acetoacetyl-CoA | 0.4 | 0.3 | 0.2 | 0.5 | 0.6 |
| L-Glutamate | 0.4 | 0.3 | 0.2 | 0.5 | 0.6 |
| 4-Aminobutanoate | 0.2 | 0.1 | 0.05 | 0.3 | 0.4 |
| Succinyl-CoA | 0.4 | 0.3 | 0.2 | 0.5 | 0.6 |
| Acetoacetate | 0.2 | 0.1 | 0.05 | 0.3 | 0.4 |
| Succinate | 0.6 | 0.5 | 0.4 | 0.7 | 0.8 |
| Succinate semialdehyde | 0.4 | 0.3 | 0.2 | 0.5 | 0.6 |
| Butanoyl-CoA | 0.4 | 0.3 | 0.2 | 0.5 | 0.6 |
| Butanoic acid | 0.2 | 0.1 | 0.05 | 0.3 | 0.4 |
| (S)-3-Hydroxy-3-methylglutaryl-CoA | 0.6 | 0.5 | 0.4 | 0.7 | 0.8 |
| Acetyl-CoA | 0.4 | 0.3 | 0.2 | 0.5 | 0.6 |
| (R)-3-Hydroxybutanoate | 0.4 | 0.3 | 0.2 | 0.5 | 0.6 |
| 4-Aminobutanoate | 0.2 | 0.1 | 0.05 | 0.3 | 0.4 |
| 2-Oxoglutarate | 0.6 | 0.5 | 0.4 | 0.7 | 0.8 |
| (S)-3-Hydroxybutanoyl-CoA | 0.4 | 0.3 | 0.2 | 0.5 | 0.6 |
| Crotonoyl-CoA | 0.4 | 0.3 | 0.2 | 0.5 | 0.6 |
| 2-Hydroxyglutarate | 0.2 | 0.1 | 0.05 | 0.3 | 0.4 |

Table S10: Perturbations in glutamate metabolism

| Metabolites | Initial Conc. | Perturbation 1 | Perturbation 2 | Perturbation 3 | Perturbation 4 |
| --- | --- | --- | --- | --- | --- |
| N-carbamoyl-L-aspartate | 0.4 | 0.3 | 0.2 | 0.5 | 0.6 |
| L-glutamine | 0.4 | 0.3 | 0.2 | 0.5 | 0.6 |
| L-glutamate | 0.4 | 0.3 | 0.2 | 0.5 | 0.6 |
| 4-aminobutanoate | 0.2 | 0.1 | 0.05 | 0.3 | 0.4 |
| 5-phosphoribosylamine | 0.4 | 0.3 | 0.2 | 0.5 | 0.6 |
| D-glucosamine-6-phosphate | 0.2 | 0.1 | 0.05 | 0.3 | 0.4 |
| D-fructose-6-phosphate | 0.6 | 0.5 | 0.4 | 0.7 | 0.8 |
| 2-oxoglutarate | 0.4 | 0.3 | 0.2 | 0.5 | 0.6 |
| 2-oxoglutaramte | 0.4 | 0.3 | 0.2 | 0.5 | 0.6 |
| succinate semialdehyde | 0.4 | 0.3 | 0.2 | 0.5 | 0.6 |
| 5-phospho-α -D-ribose-1-diphosphate | 0.4 | 0.3 | 0.2 | 0.5 | 0.6 |
| L-glutamate | 0.2 | 0.1 | 0.05 | 0.3 | 0.4 |
| 5-semialdehyde | 0.6 | 0.5 | 0.4 | 0.7 | 0.8 |
| L-aspartate | 0.4 | 0.3 | 0.2 | 0.5 | 0.6 |
| succinate | 0.4 | 0.3 | 0.2 | 0.5 | 0.6 |
